# Developing a Reliable Welfare Assessment Tool for Captive Hibernatory Bear Species

**DOI:** 10.3390/ani11113090

**Published:** 2021-10-29

**Authors:** Chloe J. Maher, Angela Gibson, Laura M. Dixon, Heather Bacon

**Affiliations:** 1 The Royal (Dick) School of Veterinary Studies, The University of Edinburgh, Edinburgh EH25 9RG, UK; 2 The Oakland Zoo, 9777 Golf Links Rd, Oakland, CA 94605, USA; agibson@oaklandzoo.org; 3 The Bear Care Group, P.O. Box 7174, Alexandria, VA 22307, USA; HBacon@uclan.ac.uk; 4 Animal Behaviour and Welfare Department, SRUC, Roslin Institute Building, Easter Bush, Edinburgh EH25 9RG, UK; laura.dixon@sruc.ac.uk; 5 The School of Veterinary Medicine, The University of Central Lancashire, Preston PR1 2HE, UK

**Keywords:** welfare assessment, *Ursidae*, animal welfare, bear, keeper ratings, zoo animal welfare

## Abstract

**Simple Summary:**

There are few welfare assessments that have been designed for captive zoo-housed species, including bears. Ideally, welfare assessment tools should be tested for reliability, feasibility and validity. This study assessed the reliability of a composite bear welfare assessment tool using keeper ratings. Two species of hibernating bears were assessed (brown and American black bears) across multiple zoos by multiple keepers. Keepers were asked to perform three assessments on the same bear. The welfare assessment tool was found to be reliable among multiple keepers assessing the same bears as well as each individual keeper performing multiple assessments over time. Further analysis showed good reliability, or unanimous ratings for each item within the assessment. This composite bear welfare assessment tool is a reliable and practical way for keepers to monitor the welfare of captive bears.

**Abstract:**

Animal welfare assessments are essential for the identification of welfare hazards and benchmarking of welfare improvements, though welfare assessments for zoo species are lacking. Bears are commonly housed in zoos but currently no composite welfare assessment tool exists for captive bears. This study describes the development of such a tool for use across hibernating bear species. A draft tool was developed using indicators derived from the literature and a modified Delphi analysis with an international group of bear keepers. A total of 18 bear keepers from 12 zoos were recruited to trial the tool on 24 brown bears and American black bears. The participating keepers assessed their bears three times across a period of nine days. Intraclass correlation coefficients analysis was used to analyse inter-, intra-rater and item reliability. The inter- and intra-rater reliability showed good to excellent levels of agreement (>0.7, *p* < 0.05). Item reliability was also assessed and showed good to excellent levels of agreement (>0.75, *p* < 0.05). The resulting bear welfare assessment is an important step in identifying and understanding challenges to bear welfare in captivity.

## 1. Introduction

Providing good animal welfare is increasingly recognised as a core activity of zoos around the world [1,2,3]. However, achieving this goal can be challenging, particularly in large carnivore species where significant welfare challenges have been reported [4,5,6]. According to ZIMS [7], there are a total of 1767 bears (*Ursidae*) (comprising brown (*Ursus arctos*), American black (*Ursus americanus*), and Asiatic black bears (*Ursus thibetanus*)) across 383 zoos worldwide; however, it can be presumed that this number is significantly higher when all species and unrecorded zoos are included. Globally there are eight species of bear, which vary greatly in size and range across multiple climates [8]. The polar bear (*Ursus maritimus*) and giant panda (*Ailuropoda melanoleuca*) are quite specialised in their diets and habitats, while other species, such as the brown bear, are more generalist with seasonally varied diets and exist within a range of habitats [8].

Welfare assessments are an important tool for determining an animal’s current welfare status, and the welfare risks of the animal’s current situation. Welfare assessments can be used to monitor animal welfare long-term [9,10] and to note any changes from a baseline. Ideally, welfare assessments should be tested for reliability, feasibility and validity [11]. Welfare assessment tools have been designed for and assessed in livestock species with the Welfare Quality^®^ protocol being the most notable (cows [12], pigs [13] and chickens [14]). Despite the extensive research regarding assessment tools for farm animals, few welfare assessments for zoo species have been designed and tested [11,15]. There has, however, been increasing interest in developing reliable, valid and robust welfare assessments for zoo species [11,16], especially of the more charismatic mammals [17].

Originally, welfare assessments focused primarily on management-based measures (MBMs) and resource-based measures (RBMs) [18,19] such as provision of food, enrichment and space. Recently, there has been a significant shift towards including animal-based (ABMs) measures, such as body condition score, and species-appropriate behaviours, as ABMs provide an actual measure of an individual’s welfare, while MBMs and RBMs only measure potential welfare risks to the animal [18,20,21]. Ideally, welfare assessments should include multiple indicators comprising all three types of measures (with a particular focus on ABMs) to obtain the best representation of an animal’s welfare and the factors that may contribute to or affect welfare [11].

To date, there has been only one study to specifically assess the welfare of captive zoo-housed bears [4], which identified welfare problems including inadequate space, inadequate substrates, and barren enclosures. There have been many studies on bear health and behaviour, both in captivity and in the wild [22,23]; however, many are focused on specific aspects of bear behaviour such as stereotypies [24]. Determining the level of welfare currently experienced by captive bears and where welfare improvements are needed should be a top priority to continue to improve our understanding of bear species and to assess the impact of management decisions on bears in captivity.

There are multiple RBMs and MBMs that have been studied in and/or are recommended for welfare assessment of bears in captivity [25]. Providing animals with choice is considered important for the improvement of welfare. A study by Ross [26] involving polar bears found that giving animals the choice to access their indoor space during the day resulted in decreased stereotypic pacing and increased play. A similar study found a decrease in abnormal behaviour and urinary cortisol in pandas when given free access to both on-exhibit and off-exhibit areas [27].

In the wild, bears can roam over very large areas [28] and can spend a significant amount of time foraging [29]. Complex and variable enclosure design has been associated with positive welfare and increased species-typical behaviours in primates [30]. Chimpanzees demonstrated similar behaviours to their wild counterparts when they were moved to a more naturalistic, variable enclosure [31]. Therefore, variable surfaces and substrate provision is recommended for bears [25] and can encourage natural foraging behaviours. The provision of areas to avoid contact with conspecifics and visitors is also recommended for bears [28,32]. Finally, the provision of environmental enrichment has been tested extensively in bears and is often found to increase foraging and reduce stereotypic behaviours [24,33,34].

There are multiple animal-based indicators of welfare that could potentially be used to assess the welfare of captive bears. Body condition score (BCS) has been used in welfare assessments of bottlenose dolphins (*Tursiops truncatus*) [15], Dorcas gazelles (*Gazella dorcas*) [35] and has been validated in African elephants (*Loxodonta africana*) [36] by measuring subcutaneous fat. A five-point BCS scale has also been validated and recommended for use in polar bears by the Association of Zoos and Aquariums [32]. Bears may also experience many health problems that affect their welfare and, therefore, measuring physical health parameters can be an important part of a welfare assessment [15,37,38]. For example: dental issues are common among bears [39], hair coat problems are seen in polar bears [32], and injuries relating to aggression from conspecifics [32] may all occur. Mobility issues such as degenerative joint disease are also common in ageing bears [39].

Finally, and arguably most important, are the behavioural indicators of welfare that can be assessed in bears. Potential indicators of positive welfare in captive bears may include foraging, climbing and play behaviours [25,28,40,41,42]. Bears spend much of their active time foraging [25], climbing [28] and play has been observed in bears of different age groups [40,41,42]. Hibernatory behaviours may also be an appropriate measure of good welfare in bears [43]. Brown, American black and Asiatic black bears are hibernatory species and reduced food intake and decreased activity may be observed during the winter months [8,22].

Traditionally, welfare assessments have been performed by trained observers with high inter-rater reliability ratings [44], though recently, focus has shifted to keeper assessments in zoos [21]. Keeper ratings were found to be reliable in cheetahs [45], rhinoceros [44], elephants [11], and gorillas [46]. To the author’s knowledge, there has only been one study involving keeper assessment of sloth bears (*Melursus ursinus*) which demonstrated that keeper ratings of bear behaviour were reliable when compared with a trained researcher [47]. Although welfare assessments using trained observers may produce a more standardised welfare assessment [44], keeper assessments can be advantageous as they can integrate a large amount of information over longer time periods [21]. Keepers may also better detect subtle changes within individuals and their behavioural responses in different situations [44] and are increasingly being used to develop welfare assessments in zoo species [48,49].

The aim of this study was to test the reliability of a welfare assessment tool that was designed for captive bears. The first objective was to assess inter- and intra-rater reliability among keepers with the aim of collecting data on multiple individual bears, across all eight species and across facilities. The second objective was to assess item reliability of each indicator of the assessment tool to determine if certain indicators are less reliable than others and should be considered for removal. This study presents results from two species of bear where sufficient data were collected for analysis.

## 2. Materials and Methods

### 2.1. Welfare Assessment Tool

Ethical approval was granted by the University of Edinburgh Veterinary Ethical Review Committee (VERC) and Human Ethical Review Committee (HERC). A pilot study was performed to evaluate the practicality of assessing selected physical and behavioural welfare indicators in a zoo bear context [50] and demonstrated practicality. Following this, veterinary and bear care specialists from animal welfare charities and zoos were contacted to provide bear welfare assessment processes, and a review of the literature describing captive and wild bear health, ecology, and behaviour was performed. This process generated 26 physical, behavioural and environmental indicators of welfare used in bears, and was drafted into a composite bear welfare assessment. In addition, a visual evaluation tool was designed to accompany the draft welfare assessment tool (see File S1 in Supplementary Materials). The visual evaluation tool provided context for some of the indicators used in the draft bear welfare assessment, e.g., descriptive scales for body condition scoring, and mobility assessment, and visual mapping of injuries or other physical abnormalities. This allowed keepers to perform a visual physical assessment of their bears and then to transform this information into a numerical score of the draft welfare assessment.

**Figure 1:**
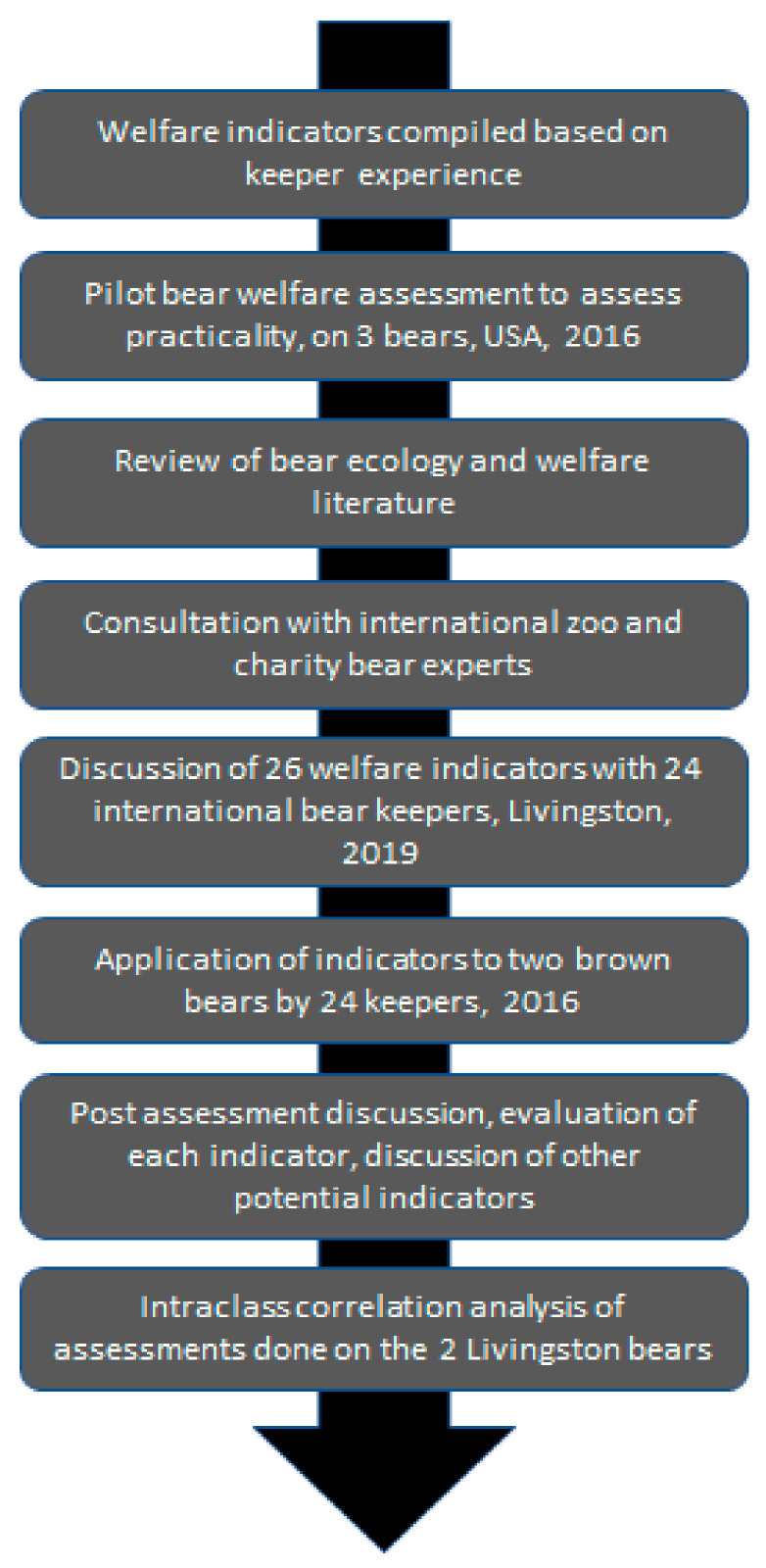
The process of development and refinement of welfare indicators taken from the literature and industry experts.

The draft welfare assessment and visual evaluation tool (see File S1 in Supplementary Materials) were introduced to 24 international bear keepers at a UK Bear Husbandry conference (Advancing Bear Care, Livingston, Scotland, 2019) as part of a modified Delphi analysis to discuss the keepers’ perspectives on the various welfare indicators, their experiences of using the assessment, and to gather their post-assessment opinions on input into any indicators not taken from the literature (Figure 1) and the keepers applied the assessment to two zoo-housed brown bears. This process involved group discussions considering ease of use of the assessment, relevance and reliability of each of the selected indicators in turn and discussion and consensus on inclusion and exclusion of potential indicators across a range of bear species. Keepers of all eight bear species were included in these discussions, and a consensus was achieved. The practical welfare assessment and discussion comprised a British and Irish Association of Zoos and Aquaria (BIAZA) accredited and certified professional development activity [51]. Based on this process three further indicators (nest building, environmental enrichment, and view out of enclosure) were added to the assessment. The final welfare assessment tool consisted of 29 welfare indicators (See Results) including indicators validated in the literature (e.g., [34]), as well as those generally accepted to be valid across species (e.g., body condition score). The maximum score for each section of the tool was as follows: physical health = 36, behaviour = 30, and environment = 21 resulting in a maximum score of 87.

### 2.2. Training Guide

Following the modified Delphi analysis of the assessment tool, a training guide was designed to aid with further data collection (see File S2 in Supplementary Materials). The assessment tool’s protocols and descriptions of each measure were outlined in a detailed and comprehensive PDF document. The training guide was designed to be used by zookeepers and included step-by-step guidelines describing how to conduct each measure and how to award scores. Visual references were also included.

### 2.3. Recruitment and Data Collection

The bear welfare assessment was posted to two closed Facebook groups—The Bear Care Group and EAZA Animal Welfare—and was also distributed through private networks using email, at the beginning of July 2020. Interested participants were emailed the training guide (see File S2 in Supplementary Materials), the welfare assessment tool, and the visual assessment tool (see File S1 in Supplementary Materials) once informed consent was given regarding the collection of personal data.

Participants were using both the assessment tool (see Results) and the visual assessment tool (see File S1 in Supplementary Materials) and were asked to read the training guide (see File S2 in Supplementary Materials) prior to beginning the assessment. Participants were then asked to perform the assessment three times per bear within the space of nine days and to return the completed assessments to the research team for analysis. Keepers were asked to score indicators based on the frequency of each behaviour that was observed over the previous seven days. General information was collected comprising the participating zookeeper’s name, their zoological institution, as well as the bear’s name, species, age, sex, social grouping, the relation to their conspecifics (e.g., siblings, mother and cub, no relation, etc.) and any changes that occurred between assessments (e.g., injuries, change in social grouping, relocation, etc.). The date and time required to complete the assessment, as well as the time of day that the assessment was performed was recorded. Data were collected from July to November 2020.

### 2.4. Data Analysis

The data were transcribed into a Microsoft Excel (2007) sheet and organised for analysis. When an assessor performed more than one assessment on the same bear, the assessment with the earliest date was labelled Day 1, the second date was labelled Day 2 and so on. In some cases, a bear was assessed by multiple assessors who each performed more than one assessment; however, the assessors did not necessarily perform their assessments on the same day. In this case, the earliest recorded assessment for each assessor was labelled as Day 1 and grouped together regardless of the date. This was the same for Day 2 and so on.

Intraclass Correlation Coefficients (ICC) was used to assess inter-observer, intra-observer and item reliability using SPSS statistical package (Version 25, IBM Corp, 2017, Armonk, NY, USA). Inter-observer reliability ICC estimates and their 95% confidence intervals were assessed per bear where there were multiple assessors based on an absolute-agreement, 2-way random-effects model. Inter-rater reliability was analysed for bears 5–8, 11–15, 22–30 and 32. Inter-rater reliability analysis could not be performed for bears 1–4, 9–10, and 17 due to these bears only having one assessor each. Bears 31 and 33–35 were also assessed for inter-rater reliability as they had two assessors each but an ICC value was not returned as one of the assessors gave all bears a full score of 87 (100%) resulting in no variance.

Intra-observer reliability was assessed per bear using an absolute-agreement, two-way mixed-effects model where the assessor(s) performed more than one assessment on the same bear. Intra-rater reliability was analysed for bears 1–4, 11–15, 17, and 22–23. Intra-rater reliability analysis could not be performed for bears 5–10, and 29–35 due to these bears being assessed only once by each assessor.

Item reliability was analysed in the case of bears that had at least two assessors that had performed at least two assessments each using an absolute-agreement, two-way random-effects model ICC analysis. Only Bears 11, 12, 13, 14, 22 and 23 met the criteria to be assessed for item reliability. Bears 12, 22 and 23 had to be excluded, as the assessors scored the bears identical scores across all assessments resulting in zero variance. ICC tests can only be run when there is variance between the scores. For B11, the indicators for Social Play and Object Play were analysed for item reliability. Object Play and Hibernatory Behaviours were the only indicators analysed in the case of B13. Object Play, Aggression, and Environmental Enrichment were the only indicators analysed in the case of B14. All other indicators for the remaining bears showed zero variance and, therefore, were excluded from analysis.

The overall mean welfare score and overall mean percentage welfare score for each bear was calculated across all assessments performed on the bear. The average score and the average percentage score of each section of the assessment tool was calculated per bear.

## 3. Results

### 3.1. Assessment Tool

The 29 welfare indicators resulting from the pilot test and modified Delphi analysis to become the assessment tool are described in Table 1. Each item was scored on a numerical scale.

### 3.2. Subjects and Study Sites

There were 24 bears in this study, comprising two species (17 Brown bears and 7 American black bears). The bears ranged in age from 6 months to 25 years (Mean = 9.171, Standard Deviation (SD) = 7.746) and there were 18 males and 6 females from 12 zoological institutions in five countries; 13 bears in the USA, 5 in the UK, 4 in Norway, 1 in Sweden and 1 in Japan. There were 18 assessors who applied the assessment to a minimum of one bear. Each bear and assessor were assigned a random number (B1–B32 for the bears and A1–A27 for assessors).

Hibernation data were missing for all three assessments of B2 by A2 as it was a young individual that had not yet experienced its first winter. There were five indicators with missing data for B13 in one assessment performed by A8 (Environmental Enrichment, Access to Indoors and Outdoors, Climate, Water Source, and Substrate). B22 had eight missing values. Weight was missing for one assessment by both A18 and A19. BCS, Forage, Access to Indoors and Outdoors, Climate, Water Source, Substrate were also missing for one of A19′s assessments. B23 had three missing values with A17 and A18 missing the indicator for Weight on one assessment each. The indicator Teeth was missing for one of A18′s assessments of B23.

### 3.3. Reliability Analysis

**Figure 2:**
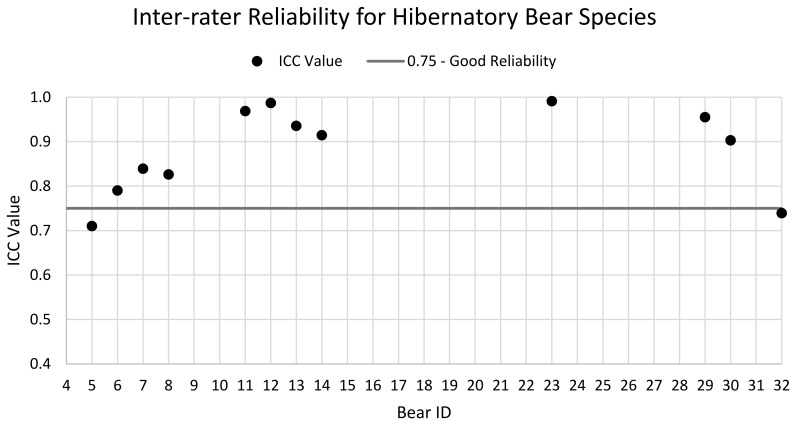
ICC values for all bears assessed for inter-rater reliability. The horizontal line sits at 0.75. Values above 0.75 are considered values of good reliability. In the case of bears with multiple assessments, the mean ICC score was calculated and depicted in the graph.

Inter-rater reliability is summarised in Figure 2. The ICC values for the bears showed good to excellent levels of agreement overall. However, 95% confidence intervals (CIs) demonstrated more variability with most values falling within the range of 0.523–1.0 (moderate to excellent) (Table 2). Bear 5 had the lowest ICC value of 0.710 and showed a poor to good 95% CIs range of 0.461–0.855. For Bears 9 and 10, only one assessment was performed by one assessor on each bear so they were removed from analysis.

**Figure 3:**
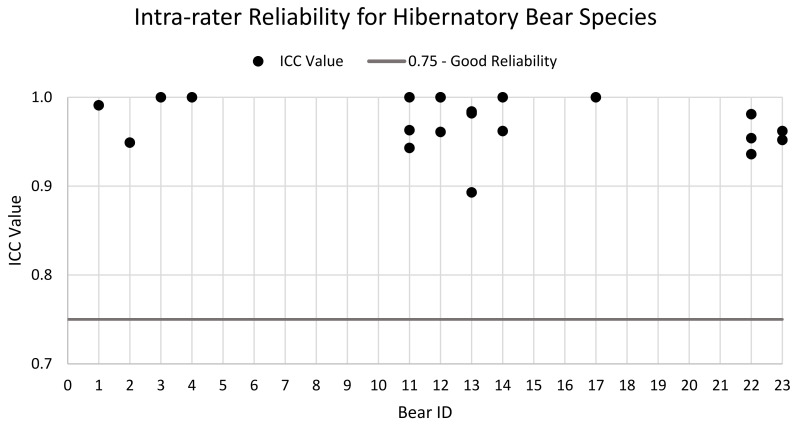
ICC values for all bears assessed for intra-rater reliability. The horizontal line sits at 0.75. Values above 0.75 are considered values of good reliability. In the case of bears with multiple assessments, the mean ICC score was calculated and depicted in the graph for each individual assessor.

Intra-rater reliability is summarised in Figure 3. The ICC values for the bears showed excellent agreement with most ICC values above 0.9. Additionally, 95% CIs demonstrated good to excellent levels of agreement with values falling between 0.791–1.0 (Table 3).

### 3.4. Item Reliability

Bears 11, 12, 13, and 14 were the only bears that could be assessed for item reliability (see Table 4). For B11, the indicator for Climb did not have enough variance in scoring to return a result. Both the Play (Social and Object) indicators returned an output. Social Play showed a moderate ICC value (ICC = 0.667) while Object Play showed an excellent ICC value (ICC = 1.0). For B13, Object Play returned an excellent ICC value (ICC = 1.0) and Hibernatory Behaviour returned a good ICC value (ICC = 0.857). Finally, for B14, the indicator for aggression did not have enough variance in scoring to return a result. Object Play returned a good ICC value (ICC = 0.889) and Environmental Enrichment returned an excellent ICC value (ICC = 1.0). ICC analysis could not be performed on the remaining 25 indicators (see Table 1) as the scoring was identical. These indicators were excluded as ICC requires variance in the scores to perform the ICC analysis.

### 3.5. Composite Assessment Scores

**Figure 4:**
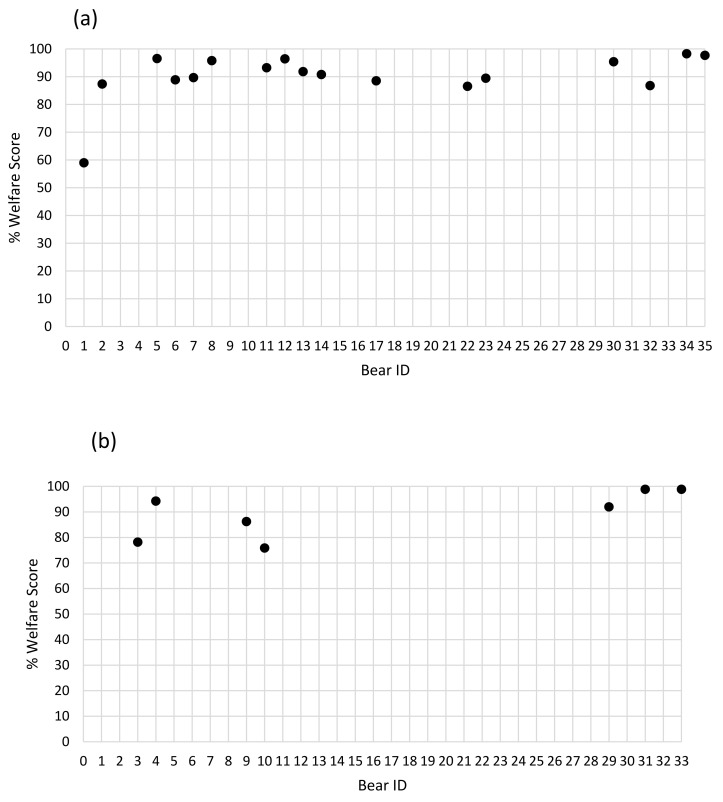
Percentage welfare score for: (**a**) brown bears and (**b**) American black bears.

The minimum score for brown bears (n = 18) was 51.33 (59%) and the maximum was 85.50 (98.28%) (mean = 78.41, standard deviation (SD) = 7.791; % mean = 90.13, SD = 8.955). The minimum score for American black bears (n = 6) was 66 (75.86%) and the maximum was 86 (98.85%) (mean = 77.57, SD = 8.162; % mean = 89.16, SD = 9.382). The percentage welfare score for each bear of both species is depicted in Figure 4.

**Figure 5:**
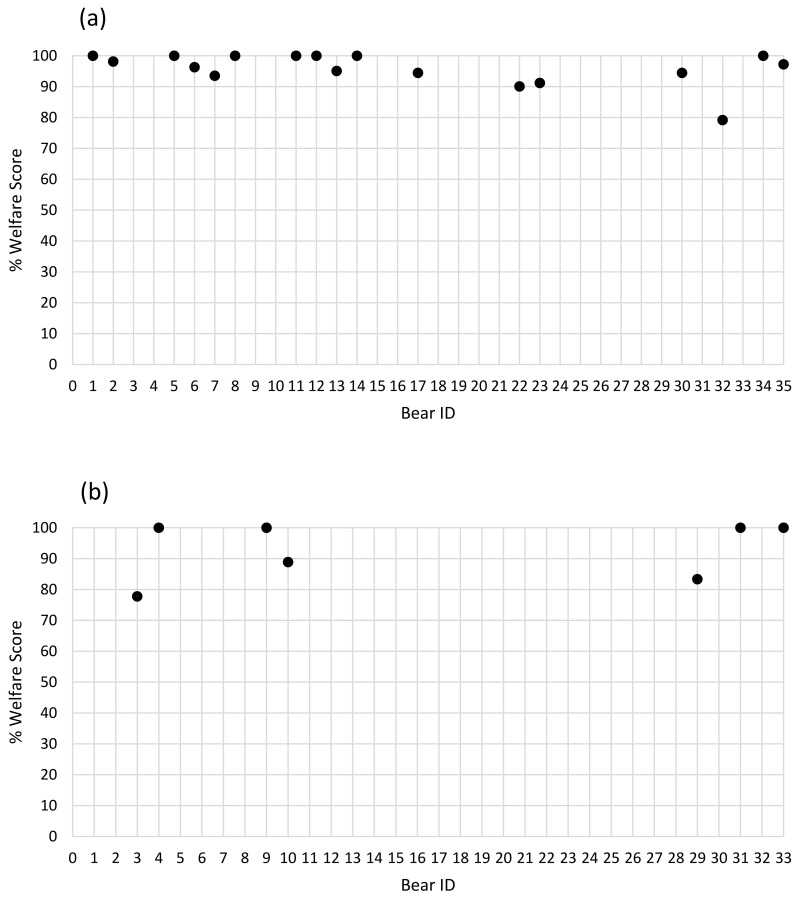

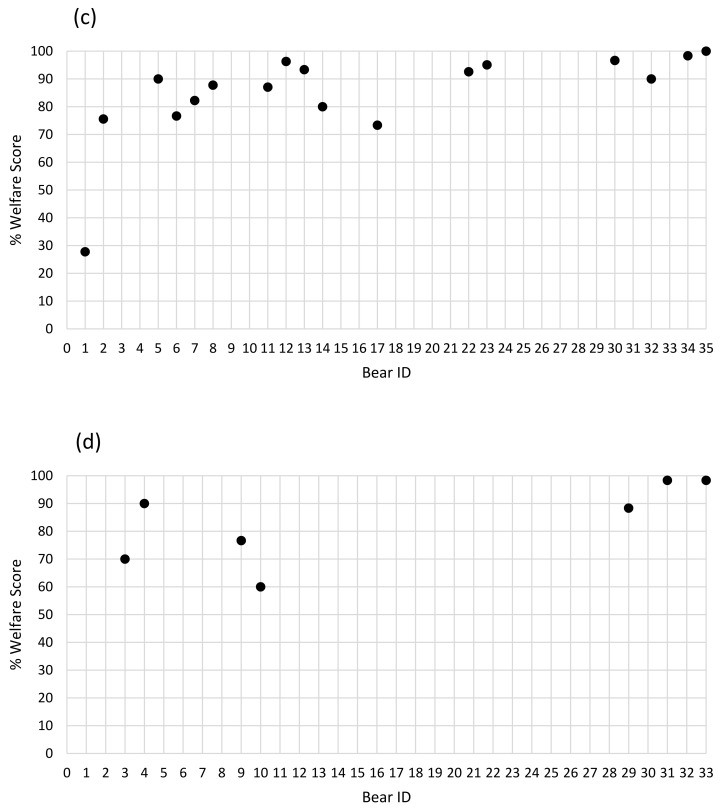

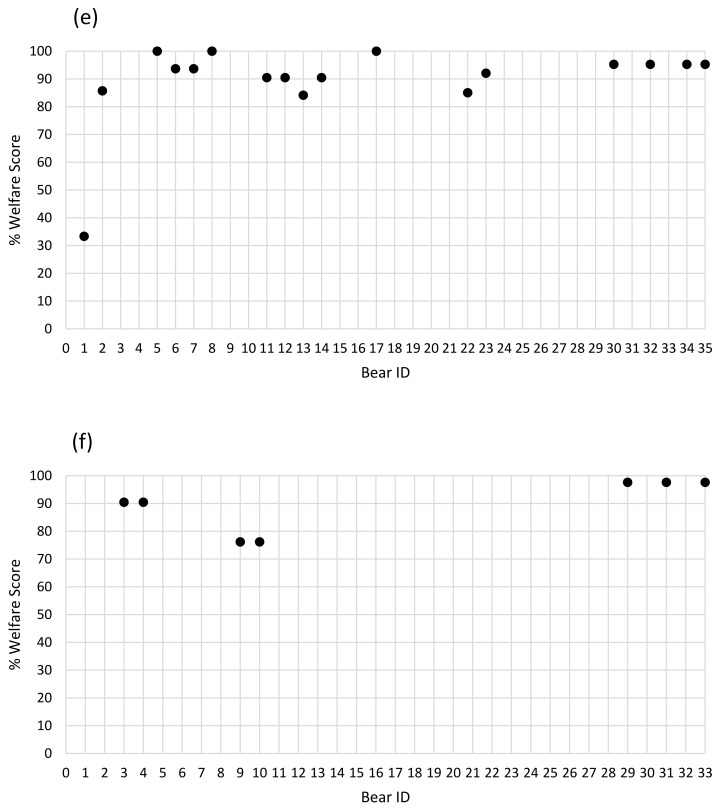
Percentage welfare scores showing: (**a**) physical health section for brown bears; (**b**) physical health section for American black bears; (**c**) behaviour section for brown bears; (**d**) behaviour section for American black bears; (**e**) environment section for brown bears; and (**f**) environment section for American black bears.

Eleven of the 24 bears were given a maximum score of 36 in the physical health section. In the environment section, three received a maximum score of 21. In the behaviour section, only one bear was given a maximum score of 30. In the physical health section, the mean score among brown bears was 34.51 (SD = 1.953; % mean = 95.86, SD = 5.426) and it was 33.43 (SD = 3.409; % mean = 92.86, SD = 9.469) among American black bears. The mean score among brown bears, in the environment section, was 18.78 (SD = 3.204; % mean = 89.41, SD = 15.256) and it was 18.79 (SD = 2.018; % mean 89.46, SD = 9.608) among American black bears. The mean score, in the behaviour section, among the brown bears was 25.13 (SD = 4.962; % mean 84.86, SD = 16.881) and 24.93 (SD = 4.392; % mean = 83.10, SD = 14.639) for American black bears. This data are summarised in Figure 5.

## 4. Discussion

The primary aim of this project was the development of a composite welfare assessment tool for captive bears, one which could be applied to hibernating species of bear. To the author’s knowledge, there is currently no welfare assessment tool that has been specifically designed to assess the welfare of bears in captivity. Such a tool is needed considering that an animal, such as a bear, has complex species-specific needs and is known to be challenging to keep in captivity [3,5,39] due to their wide-ranging ecology [5,29,52], cognitive skills [53] and complex ecology [34,54,55]. Additionally, bears are reported as experiencing a wide range of pathological health problems in the captive setting [39,56,57,58], suggesting that bear welfare is not optimal in captivity. This study demonstrates the reliability of a tool to assess bear welfare.

### 4.1. Assessment Tool

The bear welfare assessment tool is a practical and reliable tool that may be used to assess the welfare of hibernatory bear species. It is important to note that this tool was not designed to compare individuals within and across multiple zoological institutions but is rather designed to establish a baseline for each individual and to flag any changes to that individual’s welfare, for better or worse, similarly to Yon et al. [11] and Pastorino et al. [59]. Regarding the indicators used in this study, there is no good basis on which to determine the relative importance of each indicator in comparison to another. The literature review and expert consensus suggest that each of these indicators represents an important aspect of welfare for hibernatory bear species and, therefore, a low score of any one indicator should be investigated. For this reason, indicators were not weighted or ranked, as is recommended for welfare assessments intended to be used as part of a certification system [15,60,61,62].

There is growing evidence that keeper ratings of captive animals are a reliable method of monitoring animal welfare [21]. Keeper consensus is increasingly being used as a tool to contribute to the development of welfare assessments and husbandry protocols in zoo species (e.g., [48,49]). This study demonstrated similar results as previous studies assessing the reliability of keeper assessments. Keeper assessments have shown high levels of agreement when assessing elephants [11,63], chimpanzees [64], gorillas [46], cheetahs [45], rhinoceros [44], tigers [65], hyaenas [66] and sloth bears [47], to name a few. Keepers can integrate and collect detailed information about the animals under their care over a long period of time [21] providing a valuable source of information regarding an animal’s welfare, personality and behavioural repertoire, one which should be taken advantage of in future studies and continue to contribute to the evidence regarding the validity and reliability of keeper assessments.

Future steps should involve the assessment of whether species, age, sex, life history and social groupings have any significant effects on the reliability and sensitivity of the tool. Comparison of the indicators against physiological measures (e.g., cortisol levels, heart-rate monitoring, etc.) could also be investigated in the future.

Many of the indicators used in this study have recently been proposed to be valid indicators of polar bear welfare [67]. Though establishing validity of each indicator was not an objective of this study, the process and evidence for their inclusion is evidence based [4,26,34,39] and agreed by expert consensus (Figure 1). Concerns around interpretation of indicators were addressed through the educational keeper training document (see File S2 in Supplementary Materials) produced for keepers using the assessment. For example, hibernatory bears fluctuate seasonally in their weight and, therefore, BCS throughout the year. Bears will gain a significant amount of weight leading up to the period of hibernation or winter torpor [22,68,69]. It, therefore, could be argued that a bear which is “overweight” leading up to hibernation is not suffering reduced welfare but is experiencing good welfare. We mitigated this by providing examples in the keeper training guide and stating that the bear’s weight must be within the normal range for both the species and the time of year.

Certain species of bear undergo biological and behavioural seasonal changes resulting in winter torpor annually [22,70]. However, it has been found in captivity that bears will often only enter a very light winter torpor state or will not enter torpor at all [22]. Torpor or hibernation is affected by many factors in the wild, particularly by food availability, physical condition, and environmental cues, meaning management practices and zoo locations may have an impact on whether hibernation occurs [43]. Bears experiencing torpor are easily disturbed, and studies in the wild show that disturbances during torpor or ‘hibernation’ may result in metabolic derangements and activity for several days [43]. For zoo bears experiencing daily disturbances due to husbandry routines and visitor activity, achieving torpor may not be possible, even when bears demonstrate significant behavioural inactivity (e.g., [71]). Allowing bears to hibernate requires the provision of appropriate environmental provisions and a secluded and undisturbed environment [72]. Therefore, winter torpor behaviours have been suggested to be important for bear welfare and keepers from zoos who allow their bears to hibernate felt strongly that it benefitted the bears both physically and mentally [73].

### 4.2. Inter- and Intra-Rater Reliability

ICC values showed good to excellent agreement for both inter- and intra-rater reliability. Of the thirteen bears (and their assessors) that were assessed for inter-rater reliability, ten of the bears’ ICC values sit above 0.75, the value considered an indication of good reliability, as discussed by Koo and Li [74].

Only two of the bears (B5 and B32) analysed for inter-rater reliability sit below 0.75 with ICC values of 0.710 and 0.739, respectively. These lower reliability scores can be caused by different factors. B5 was assessed by A4, A5, and A6 while B32 was assessed by A26 and A27. The variability may be due to the assessors themselves; however, in both cases these assessors also assessed other bears (these bears had high ICC values) suggesting that the variation is not necessarily with the assessors’ understanding and interpretation of the assessment tool, but it is more likely to be with the bear being assessed. Each bear was assessed three times by each assessor, while there were no reported significant changes in the bear’s health and environment within the short timeframe of the assessments, the bear’s behaviour may have shown variation across the repeated measurements, resulting in the lower reliability scores. Behavioural variation could occur as a response to different assessors and is sometimes included as a measure in welfare assessments, e.g., [75]. Further research is needed to analyse behavioural responses to different keepers and whether these may influence bear welfare assessments, as well as long-term studies to determine if variability in the bear’s behaviour across time and seasons may result in lower ICC values and, therefore, lower reliability.

As this study was conducted remotely, none of the researchers were present during any of the keeper’s assessments and the method of data collection was not standardised (e.g., time of day, specific days, etc.). To optimise practicality, keepers were asked to perform the assessments whenever they had time among their other duties. The low inter-observer reliability scores of the assessments performed on B5 and B32 could possibly be explained by the assessors having different levels of familiarity with the bears or the bears behaving or responding differently in the presence of different keepers.

Intra-rater reliability was excellent (>0.75) for all assessors. It is expected that there will always be less variability among the same assessor’s scores then there would be across multiple assessors as multiple assessors will show more variability in their scoring when compared with each other [76]. In this study, the assessors were asked to perform three assessments within a nine-day period to limit the influences of seasonality of husbandry changes on bear behaviour and health causing variation in scoring. It is reasonable to assume that, due to this short timeframe, assessors may remember some of their scores from the previous assessment; however, remembering all 29 is unlikely, especially for assessors rating multiple bears. Future studies could increase the time elapsed between assessments, but this may introduce artificial variation, as bear behaviour and health are more likely to change over time and, thus, generate more variable ratings. However, since this study has demonstrated the reliability of this assessment tool, keepers could use the assessment seasonally to monitor changes in the health and behaviour of the bears in their care and can trust that the tool is accurately reflecting these changes.

### 4.3. Item Reliability

Only four bears (B11, B12, B13 and B14) and only four indicators (Social Play, Object Play, Hibernatory Behaviours and Environmental Enrichment) could be assessed at least once for reliability. Social Play was the only indicator that received a reliability value < 0.75. Because ICC analysis requires variance in the inputted values to return a result, the indicators for most of the bears, other than the ones listed above, did not show enough variance or enough data points to be analysed for reliability and this in itself indicates a high level of item reliability across different assessors, and suggests that individual items within the composite assessment are reliable between different bears and different assessors.

### 4.4. Welfare Scores

There have been specific welfare assessments designed for American black bears and polar bears through the WelfareTrak^®^ application created by Whitham and Wielebnowski [77] and there has been one welfare assessment of multiple species of bears [4] of which the primary aim was to identify welfare issues in Polish zoos. The study described in this paper, however, is the first detailing a welfare assessment designed to apply to all species of hibernating bear and to be used across multiple institutions by keepers. The overall welfare scores of all bears were quite high, above 75%, except for a single brown bear which scored 59%. Though it is difficult to determine at which percentage a welfare score should be considered good or excellent, it is clear in this study that most of the bears were scored quite high in general. It is more likely that these high welfare scores are due to more ‘welfare-friendly’ keepers and zoos being interested in participating in this study than an indication of overall high levels of welfare among captive bears. Therefore, the results of this study in terms of the welfare score of captive bears must be interpreted with caution as they may not be an accurate example of the general captive population.

The general scoring for the physical health and environment sections were consistently high with some keepers giving the bears in their care the full cumulative score for those sections. The behaviour section, however, showed more variation in scoring, though only one brown bear scored less than half the score available for that section (B1) (Figure 2). Since inter- and intra-rater reliability values were good overall, and the physical health and environment sections had consistent scores among keepers, the variation in the behaviour section is likely due to the individual bear’s behavioural repertoires. Play has been observed in both adult and juvenile brown bears [41,42] and in American black bears [40]. Play has been proposed as a potential indicator of positive welfare; however, the relationship between play and welfare is highly complex [78,79]. Play has been known to both decrease and increase when animals are experiencing stressful conditions [78]. Blois-Heulin et al. [79] found, in horses and macaques, that the individuals that played the most also exhibited the most stereotypic behaviours suggesting that play is not a reliable positive welfare indicator. Heesen et al. [80], on the other hand, suggested that play involves a social element of cooperation which is beneficial for animals. Similar to hibernation, play in bears requires more research.

Stereotypy is a commonly used indicator of negative welfare [11,33,34]. Stereotypies are considered repetitive, invariant behaviours [81] and may be a coping mechanism resulting from sub-optimal environments [82]. Bears appear to be particularly prone to stereotypies in captivity [5]. Large home range sizes in the wild and foraging/hunting motivations have been correlated with pacing stereotypies [6]. High levels of faecal cortisol have also been correlated with higher proportions of stereotypy in giant pandas [83] and polar bears [34]. There is much debate within the literature regarding the function or purpose of stereotypies; however, it is still widely considered to be a sign of negative welfare [82]. Stereotypies are not necessarily indicative of an animal’s current welfare state as they can often persist after an animal has been moved to a more appropriate environment [11,82] and, thus, should be interpreted with caution. Alternative methodologies, such as counting behavioural frequency, allows keepers to integrate a large amount of information about the bear’s behavioural repertoire particularly regarding behaviours that are considered rare [21] and may be more appropriate for monitoring behaviour in the long-term, but can result in a less standardised method of data collection. This makes it difficult to eliminate certain mitigating factors such as keepers influencing each other’s assessment scores and seasonal changes in a bear’s behavioural repertoire potentially flagging an increase or decrease in a certain behaviour as a welfare concern when this may not be the case. The multiple factors that may influence animal behaviour is why a holistic approach with multiple indicators of welfare is necessary to obtain a clearer picture of an animal’s welfare. However, this study does raise questions about the behavioural aspects of welfare of bears in the captive environment.

Bears have a large range of behaviours [84], which may differ between individuals resulting in the variation seen in this study. Other factors that may contribute to this variation is early life experience, (which affected breeding behaviours in cheetahs [45]), inadequate space (bears are naturally wide-ranging [5,8], which has been correlated with stereotypic pacing [29]) and behavioural frustration (Tan et al. [55] contributed both locomotory and oral stereotypies in sun bears to frustration due to inadequate space and environmental complexity). Social groupings may also impact behaviour, as seen in a study where a male bear began to perform stereotypic behaviour when a previously receptive female in oestrus began to reject his advances [85].

The welfare scores generated using the assessment tool are useful in showing the generally good welfare enjoyed by zoo-housed bears in this study (within the limitations discussed). However, the behaviour scores support the existing literature that behavioural aspects of welfare are an area of concern in captive bears (even among a sample of what is likely to be a cohort of welfare-focused bear keepers as in this study). The behavioural scores were low when compared with the high scores for environmental indicators suggesting that providing good environmental provisions may not be enough to result in good behavioural welfare and there are other factors that may be required to achieve this. One of these factors may be a good keeper-animal relationship for which there is evidence, in other species, that such a relationship may enhance the welfare of zoo animals [86,87] and would be an important factor to investigate in future studies.

## 5. Conclusions

This welfare assessment tool is the first of its kind to be designed for hibernating bears and was intended to be simple, easy to use and practical, allowing keepers to incorporate it into their regular husbandry tasks.

This study demonstrates the successful assessment of reliability of a composite welfare assessment tool for captive bears. Further research should aim to understand the long-term application of the tool across various zoological institutions worldwide and the welfare of the bears in this assessment. Ultimately, it is hoped that this welfare assessment tool may be successfully utilized by keepers to benchmark and monitor the welfare of captive bears and contribute to improving their welfare.

## Figures and Tables

**Table 1:** Welfare assessment indicators with their corresponding definitions and scoring system.

Indicator	Description	Score
Physical Health (n = 12)		
Weight	Normal range for species and season	3
	Outside of normal range	1
Body Condition Score ^1^	Score of 4, 5 or 6	3
	Score of 1, 2, 3, 7, 8, or 9	1
Visual Assessment of eyes, nose, teeth, skin, haircoat, claws, footpads, and injuries/wounds	No abnormality present	3
	Any abnormality present	1
Mobility Hindlimb Assessment ^1^	Mobility score of 1	3
	Mobility score of 2	2
	Mobility score of 3–4	1
	Mobility score of 5–6	0
Cincinnati Gait Assessment ^1^	0–10	3
	11–40	2
	41–60	1
	61+	0
Behaviour (n = 10)		
Foraging	Bear searches for food throughout the environment	Score 3 if behavior observed daily, score 2 if 2–3× per week, 1 if less than twice a week, 0 if never observed
Climbing	Bear uses structures or trees within the enclosure to climb off the ground	
Social Play	Bear interacts positively with other bears, including wrestling/swatting/interacting with soft, open mouth	
Object Play	Bear interacts positively and non-repetitively with an object	
Nest Building	Bear gathers provided materials together into a pile to rest on during the day	
Aggression	Bear displays aggressive behaviour including growling, roaring, charging or swatting towards another bear or a human	Score 0 if behaviour is observed daily, 1 if 2–3× per week, 2 if less than twice a week, 3 if never observed
Abnormal Behaviour/Stereotypy	Bear displays repetitive head-swaying, pacing, rocking, pouncing, or object rolling/placing behaviour	
Feeding	Bear is fed a seasonally variable diet according to behaviours shown	3
	Bear’s diet remains generally stable but may change once or twice per year	2
	Bear is fed a standard ration through the year or fed to maintain a consistent bodyweight	1
Hibernatory/Winter Torpor Behaviours	Bear has displayed seasonal dormancy/torpor and been encouraged to sleep through the winter	3
	Bear has slowed down but only slept for short intermittent periods through the winter	2
	Bear has slowed down but remained awake through the year	1
	Bear has been routinely exhibited and not encouraged to sleep	0
Environmental Enrichment	Enrichment is provided daily through an enrichment calendar, type and time of presentation is varied, enrichment may be incorporated into normal husbandry or a training programme	3
	Enrichment is provided 2–3× per week, though type and time may only vary occasionally	2
	Enrichment is provided less than twice a week	1
	Enrichment is never provided	0
Environment (n = 7)		
Access to Indoor and Outdoor areas	24 h (except enclosure servicing)	3
	Access to both areas > 18 h/day	2
	Access limited to one area for at least 7 h	1
Local Temperature Range/Climate	Species endemic to local area or endemic to similar climatic conditions	3
	Environment is broadly temperate, arctic or tropical in line with species ecology	2
	Environmental climate is dissimilar to species natural environment	1
Water Source for Bathing	Present	3
	Absent	1
Surfaces and Substrates	Naturalistic and varied substrates to enable a range of behaviours including digging, nesting, bathing and foraging	3
	Naturalistic substrates are provided but behavioural opportunities are limited	2
	Enclosure contains a mixture of artificial and natural surfaces (e.g., primarily artificial but with a digging pit and/or log piles)	1
	Surfaces and substrates cannot be manipulated and provide no behavioural opportunities	0
View Out of Enclosure	Enclosure consists of glass windows or fencing which allows the bear to see surrounding area outside enclosure	3
	Enclosure is surrounded by high walls with only ‘open’ area utilised as a visitor viewing area	1
Cover and Privacy	Enclosure offers varied topography, visual barriers and limited viewing	3
	Enclosure offers some privacy	2
	Animal is on view to visitors without cover	1
Spatial Complexity	Enclosure offers environmental complexity and a range of behavioural opportunities including climbing, bathing, foraging and exploration over a variety of 3D spaces	3
	Enclosure offers some environmental complexity and some behavioural opportunities but this could be improved	2
	Enclosure is simple with limited opportunities to display natural behaviours	1
	Enclosure is barren with no complexity	0

^1^ See File S1 in Supplementary Materials for the Body Condition Score, the Mobility Hindlimb Assessment and the Cincinnati Gait Assessment tables.

**Table 2:** Level of agreement between assessors for each bear assessed for inter-rater reliability.

Bear ID	Day	ICC Value	95% Confidence Interval
5	1	0.710	0.461–0.855
6	1	0.790	0.610–0.895
7	1	0.839	0.702–0.919
8	1	0.826	0.679–0.912
11	1	0.943	0.894–0.971
	2	0.963	0.932–0.982
	3	1.0	1.0
12	1	0.961	0.928–0.980
	2	1.0	1.0
	3	1.0	1.0
13	1	0.964	0.929–0.983
	2	0.877	0.774–0.938
	3	0.965	0.935–0.982
14	1	0.872	0.728–0.940
	2	0.957	0.909–0.980
22	1	0.983	0.966–0.993
	2	1.0	1.0
23	1	0.982	0.960–0.992
	2	1.0	1.0
29	1	0.955	0.905–0.979
30	1	0.903	0.794–0.954
32	1	0.739	0.447–0.877

**Table 3:** Level of agreement between assessors for each bear assessed for intra-rater reliability.

Assessor ID	Bear ID	ICC Value	95% Confidence Interval
A1	1	0.991	0.983–0.995
A2	2	0.949	0.906–0.975
A3	3	1.0	1.0
A3	4	1.0	1.0
A8	11	0.943	0.894–0.971
A8	12	0.961	0.928–0.980
A8	13	0.893	0.791–0.950
A9	11	1.0	1.0
A9	12	1.0	1.0
A9	13	0.982	0.968–0.991
A10	11	0.963	0.932–0.982
A10	12	1.0	1.0
A10	13	0.984	0.971–0.992
A11	14	0.962	0.919–0.982
A12	14	1.0	1.0
A14	17	1.0	1.0
A17	22	0.936	0.880–0.968
A17	23	0.952	0.911–0.976
A18	22	0.954	0.898–0.979
A18	23	0.962	0.917–0.983
A19	22	0.981	0.954–0.992

**Table 4:** Item Reliability for indicators including their 95% Confidence Intervals.

Bear ID	Bear Species	Assessors	Indicators	ICC Value	95% Confidence Interval
11	Eurasian brown bear	A8, A9 and A10 (3 assessments each)	Social Play	0.667	−4.878–0.991
			Object Play	1.0	*
13	Eurasian brown bear	A8, A9 and A10 (3 assessments each)	Object Play	1.0	*
			Hibernatory Behaviour	0.857	−0.045–0.996
14	Eurasian brown bear	A11 and A12 (2 assessments each)	Object Play	0.889	−3.414–1.0
			Environmental Enrichment	1.0	*

* 95% Confidence Intervals were not returned in these cases.

## Data Availability

The data presented in this study are available on request from the corresponding author. The data are not publicly available due to privacy reasons.

## Supplementary Materials

The following are available online at https://www.mdpi.com/article/10.3390/ani11113090/s1, File S1: Visual Assessment Tool, File S2: Training Guide.

## References

[1] Mellor D.J., Hunt S., Gusset M. (2015). Caring for Wildlife: The World Zoo and Aquarium Animal Welfare Strategy.

[2] Sherwen S., Hemsworth L.M., Beausoleil N.J., Embury A., Mellor D.J. (2018). An Animal Welfare Risk Assessment Process for Zoos. Animals.

[3] Blackett T.A., McKenna C., Kavanagh L., Morgan D.R. (2017). The welfare of wild animals in zoological institutions: Are we meeting our duty of care?. Int. Zoo Yearb..

[4] Maślak R., Sergiel A., Bowles D., Paśko L. (2016). The Welfare of Bears in Zoos: A Case Study of Poland. J. Appl. Anim. Welf. Sci..

[5] Clubb R., Mason G. (2003). Captivity effects on wide-ranging carnivores. Nature.

[6] Kroshko J., Clubb R., Harper L., Mellor E., Moehrenschlager A., Mason G. (2016). Stereotypic route tracing in captive Carnivora is predicted by species-typical home range sizes and hunting styles. Anim. Behav..

[7] (2020). Species360 Zoological Information Management System (ZIMS). https://www.species360.org/about-us/contact-us/.

[8] Garshelis D.L., Lindburg D., Baragona K. (2004). Chapter 4: Variation in Ursid Life Histories, Is There an Outlier?. Giant Pandas: Biology and Conservation.

[9] Barber J.C. (2009). Programmatic approaches to assessing and improving animal welfare in zoos and aquariums. Zoo Biol..

[10] Department for Environment, Food, and Rural Affairs (DEFRA) (2012). Chapter 4: Animal welfare and its assessment in zoos. DEFRA, Zoos Expert Committee Handbook.

[11] Yon L., Williams E., Harvey N.D., Asher L. (2019). Development of a behavioural welfare assessment tool for routine use with captive elephants. PLoS ONE.

[12] De Graaf S., Ampe B., Tuyttens F. (2017). Assessing dairy cow welfare at the beginning and end of the indoor period using the Welfare Quality^®^ protocol. Anim. Welf..

[13] Temple D., Dalmau A., de la Torre J.L.R., Manteca X., Velarde A. (2011). Application of the Welfare Quality^®^ protocol to assess growing pigs kept under intensive conditions in Spain. J. Vet. Behav..

[14] Buijs S., Ampe B., Tuyttens F.A.M. (2017). Sensitivity of the Welfare Quality^®^ broiler chicken protocol to differences between intensively reared indoor flocks: Which factors explain overall classification?. Animal.

[15] Clegg I., Borger-Turner J., Eskelinen H. (2015). C-Well: The development of a welfare assessment index for captive bottlenose dolphins (Tursiops truncatus). Anim. Welf..

[16] Kuhar C.W., Stoinski T.S., Lukas K.E., Maple T. (2006). Gorilla Behavior Index revisited: Age, housing and behavior. Appl. Anim. Behav. Sci..

[17] Melfi V.A. (2009). There are big gaps in our knowledge, and thus approach, to zoo animal welfare: A case for evidence-based zoo animal management. Zoo Biol..

[18] Main D.C.J., Whay H.R., Leeb C., Webster A.J.F. (2007). Formal Animal-Based Welfare Assessment in UK Certification Schemes. Anim. Welf..

[19] Main D.C.J. (2009). Application of Welfare Assessment to Commercial Livestock Production. J. Appl. Anim. Welf. Sci..

[20] Farm Animal Welfare Council (FAWC) (2009). Five Freedoms. https://webarchive.nationalarchives.gov.uk/20121010012427/http://www.fawc.org.uk/freedoms.htm.

[21] Whitham J.C., Wielebnowski N. (2009). Animal-based welfare monitoring: Using keeper ratings as an assessment tool. Zoo Biol..

[22] Kim M., Jeong D., Yeon S. (2020). Hibernation behaviour and ethogram of captive Asiatic black bear (*Ursus thibetanus*). Veterinární Med..

[23] Robbins C.T., Lopez-Alfaro C., Rode K., Tøien Ø., Nelson O.L. (2012). Hibernation and seasonal fasting in bears: The energetic costs and consequences for polar bears. J. Mammal..

[24] Forthman D.L., Elder S.D., Bakeman R., Kurkowski T.W., Noble C.C., Winslow S.W. (1992). Effects of feeding enrichment on behavior of three species of captive bears. Zoo Biol..

[25] Law G., Reid A. (2010). Enriching the lives of bears in zoos. Int. Zoo Yearb..

[26] Ross S.R. (2006). Issues of choice and control in the behaviour of a pair of captive polar bears (*Ursus maritimus*). Behav. Process..

[27] Owen M.A., Swaisgood R.R., Czekala N.M., Lindburg D.G. (2005). Enclosure choice and well-being in giant pandas: Is it all about control?. Zoo Biol..

[28] Sergiel A., Maślak R., Gardocka T., Gruszczyńska A., Maślak R., Sergiel A. (2014). Part II: The Welfare of Zoo Animals, Chapter 4: The welfare of captive bears. The Welfare of Animals in Zoos and EU Legal Standards.

[29] Clubb R., Mason G.J. (2007). Natural behavioural biology as a risk factor in carnivore welfare: How analysing species differences could help zoos improve enclosures. Appl. Anim. Behav. Sci..

[30] Hosey G.R. (2005). How does the zoo environment affect the behaviour of captive primates?. Appl. Anim. Behav. Sci..

[31] Ross S.R., Schapiro S.J., Hau J., Lukas K.E. (2009). Space use as an indicator of enclosure appropriateness: A novel measure of captive animal welfare. Appl. Anim. Behav. Sci..

[32] AZA Bear Taxon Advisory Group (2009). Polar Bear (Ursus maritimus) Care Manual.

[33] Carlstead K., Seidensticker J., Baldwin R. (1991). Environmental enrichment for zoo bears. Zoo Biol..

[34] Shepherdson D., Lewis K.D., Carlstead K., Bauman J., Perrin N. (2013). Individual and environmental factors associated with stereotypic behavior and fecal glucocorticoid metabolite levels in zoo housed polar bears. Appl. Anim. Behav. Sci..

[35] Salas M., Manteca X., Abáigar T., Delclaux M., Enseñat C., Martínez-Nevado E., Quevedo M., Fernández-Bellon H. (2018). Using Farm Animal Welfare Protocols as a Base to Assess the Welfare of Wild Animals in Captivity—Case Study: Dorcas Gazelles (*Gazella dorcas*). Animals.

[36] Morfeld K.A., Lehnhardt J., Alligood C., Bolling J., Brown J.L. (2014). Development of a Body Condition Scoring Index for Female African Elephants Validated by Ultrasound Measurements of Subcutaneous Fat. PLoS ONE.

[37] AZA Bear Taxon Advisory Group (2019). Sun and Sloth Bear Care Manual.

[38] Mononen J., Møller S.H., Hansen S., Hovland A., Koistinen T., Lidfors L., Malmkvist J., Vinke C., Ahola L. (2012). The development of on-farm welfare assessment protocols for foxes and mink: The WelFur project. Anim. Welf..

[39] Bourne D.C., Cracknell J.M., Bacon H.J. (2010). Veterinary issues related to bears (Ursidae). Int. Zoo Yearb..

[40] Henry J.D., Herrero S.M. (1974). Social Play in the American Black Bear: Its Similarity to Canid Social Play and an Examination of its Identifying Characteristics. Integra. Comp. Biol..

[41] Fagan R., Fagan J. (1990). Play behavior of brown bears (*Ursus arctos*) and human presence at Pack Creek, Admiralty Island, Alas-ka. Bears: Their Biology and Management, International Conference on Bear Research and Management.

[42] Fagen R., Fagan J. (2004). Juvenile survival and benefits of play behaviour in brown bears, Ursus arctos. Evol. Ecol. Res..

[43] Evans A.L., Singh N.J., Friebe A., Arnemo J.M., Laske T.G., Fröbert O., Swenson J.E., Blanc S. (2016). Drivers of hibernation in the brown bear. Front. Zool..

[44] Carlstead K., Mellen J., Kleiman D.G. (1999). Black Rhinoceros (*Diceros bicornis*) in U.S. Zoos: I. Individual Behavior Profiles and Their Relationship to Breeding Success. Zoo Biol..

[45] Wielebnowski N.C. (1999). Behavioral differences as predictors of breeding status in captive cheetahs. Zoo Biol..

[46] Less E.H., Kuhar C.W., Dennis P.M., Lukas K.E. (2012). Assessing inactivity in zoo gorillas using keeper ratings and behavioral data. Appl. Anim. Behav. Sci..

[47] Khadpekar Y., Whiteman J.P., Durrant B.S., Owen M.A., Prakash S. (2018). Approaches to studying behavior in captive sloth bears through animal keeper feedback. Zoo Biol..

[48] Rose P., Brereton J.E., Gardner L. (2016). Developing flamingo husbandry practices through workshop communication. J. Zoo Aqua. Res..

[49] Williams E., Carter A., Hall C., Bremner-Harrison S. (2019). Exploring the relationship between personality and social interactions in zoo-housed elephants: Incorporation of keeper expertise. Appl. Anim. Behav. Sci..

[50] Gibson A. Assessing Quality of life in bears. Proceedings of the Advancing Bear Care.

[51] Bacon H., Gibson A. Bear Welfare Assessment. Proceedings of the Advancing Bear Care.

[52] Bauer E., Babitz M., Boedeker N., Hellmuth H. (2013). Approaches to understanding and managing pacing in sloth bears in a zo-ological setting. Int. J. Comp. Psychol..

[53] Tabellario S., Babitz M.A., Bauer E.B., Brown-Palsgrove M. (2020). Picture recognition of food by sloth bears (*Melursus ursinus*). Anim. Cogn..

[54] Tan H., Ong S., Langat G., Bahaman A., Sharma R., Sumita S. (2013). The influence of enclosure design on diurnal activity and stereotypic behaviour in captive Malayan Sun bears (*Helarctos malayanus*). Res. Vet. Sci..

[55] McGowan R.T.S., Robbins C.T., Alldredge J.R., Newberry R.C. (2010). Contrafreeloading in grizzly bears: Implications for captive foraging enrichment. Zoo Biol..

[56] Maślak R., Sergiel A., Hill S.P. (2013). Some aspects of locomotory stereotypies in spectacled bears (*Tremarctos ornatus*) and changes in behavior after relocation and dental treatment. J. Vet. Behav..

[57] Föllmi J., Steiger A., Walzer C., Robert N., Geissbühler U., Doherr M., Wenker C. (2007). A scoring system to evaluate physical condition and quality of life in geriatric zoo mammals. Anim. Welf..

[58] Kitchener A. (2004). The problems of old bears in zoos. Int. Zoo News.

[59] Pastorino G.Q., Christodoulides Y., Curone G., Pearce-Kelly P., Faustini M., Albertini M., Preziosi R., Mazzola S.M. (2017). Behavioural Profiles of Brown and Sloth Bears in Captivity. Animals.

[60] Spoolder H., de Rosa G., Hörning B., Waiblinger S., Wemelsfelder F. (2003). Integrating Parameters to Assess On-farm Welfare. Anim. Welf..

[61] Botreau R., Gaborit M., Veissier I. Applying Welfare Quality^®^ Strategy to Design a Welfare Assessment Tool for Foxes and Mink Farms. Proceedings of the Xth International Scientific Congress in Fur Animal Production.

[62] Barnard S., Pedernera C., Candeloro L., Ferri N., Velarde A., Villa P.F.D. (2016). Development of a new welfare assessment protocol for practical application in long-term dog shelters. Vet. Rec..

[63] Bonaparte-Saller M., Mench J.A. (2018). Assessing the dyadic social relationships of female african (*Loxodonta africana*) and asian (*Elephas maximus*) zoo elephants using proximity, tactile contact, and keeper surveys. Appl. Anim. Behav. Sci..

[64] King J.E., Landau V.I. (2003). Can chimpanzee (*Pan troglodytes*) happiness be estimated by human raters?. J. Res. Personal..

[65] Phillips C.J., Tribe A., Lisle A., Galloway T.K., Hansen K. (2017). Keepers’ rating of emotions in captive big cats, and their use in determining responses to different types of enrichment. J. Vet. Behav..

[66] Gosling S.D. (1998). Personality dimensions in spotted hyenas (*Crocuta crocuta*). J. Comp. Psychol..

[67] Skovlund C., Kirchner M., Moos L., Alsted N., Manteca X., Tallo-Parra O., Stelvig M., Forkman B. (2021). A critical review of animal-based welfare indicators for polar bears (*Ursus maritimus*) in zoos: Identification and evidence of validity. Anim. Welf..

[68] Hashimoto Y., Yasutake A. (1999). Seasonal changes in body weight of female Asiatic black bears under captivity. Mammal Study.

[69] Hilderbrand G.V., Jenkins S.G., Schwartz C.C., Hanley T.A., Robbins C.T. (1999). Effect of seasonal differences in dietary meat intake on changes in body mass and composition in wild and captive brown bears. Can. J. Zool..

[70] Soriano A.I., Vinyoles D., Maté C. (2017). Abnormales Verhalten in Zwei Gefangen Grizzlybär-Weibchen (*Ursus arctos linnaeus*, 1758): Einzelne Unterschiede Und Saisonale Variationen. Zool. Gart..

[71] Fernandez E.J., Yoakum E., Andrews N. (2020). Seasonal and daily activity of two zoo-housed grizzly bears (*Ursus arctos horri-bilis*). J. Zool. Bot. Gard..

[72] Itoh K., Ide K., Kojima Y., Terada M. (2010). Hibernation exhibit for Japanese black bear Ursus thibetanus japonicus at Ueno Zoological Gardens. Int. Zoo Yearb..

[73] Forde J. The Development and Benefits of Captive Torpor in European Brown Bears. Proceedings of the Advancing Bear Care.

[74] Koo T.K., Li M.Y. (2016). A Guideline of Selecting and Reporting Intraclass Correlation Coefficients for Reliability Research. J. Chiropr. Med..

[75] Main D.C.J., Webster A.J.F., Green L.E. (2001). Animal Welfare Assessment in Farm Assurance Schemes. Acta Agric. Scand. Sect. A Anim. Sci..

[76] de Vet H.C., Terwee C.B., Knol D.L., Bouter L. (2006). When to use agreement versus reliability measures. J. Clin. Epidemiol..

[77] Whitham J.C., Wielebnowski N. (2013). New directions for zoo animal welfare science. Appl. Anim. Behav. Sci..

[78] Held S.D., Špinka M. (2011). Animal play and animal welfare. Anim. Behav..

[79] Blois-Heulin C., Rochais C., Camus S., Fureix C., Lemasson A., Lunel C., Bezard E., Hausberger M. (2015). Animal Welfare: Could Adult Play be a False Friend?. Anim. Behav. Cogn..

[80] Heesen R., Genty E., Rossano F., Zuberbühler K., Bangerter A. (2017). Social play as joint action: A framework to study the evolution of shared intentionality as an interactional achievement. Learn. Behav..

[81] Mason G.J. (1991). Stereotypies: A critical review. Anim. Behav..

[82] Mason G.J., Latham N.R. (2004). Can’t Stop, Won’t Stop: Is Stereotypy a Reliable Animal Welfare Indicator?. Anim. Welf..

[83] Liu D., Wang Z., Tian H., Yu C., Zhang G., Wei R., Zhang H. (2003). Behavior of giant pandas (*Ailuropoda melanoleuca*) in captive conditions: Gender differences and enclosure effects. Zoo Biol..

[84] Hüber D. (2010). Rehabilitation and reintroduction of captive-reared bears: Feasibility and methodology for European brown bears Ursus arctos. Int. Zoo Yearb..

[85] Fischbacher M., Schmid H. (1999). Feeding Enrichment and Stereotypic Behavior in Spectacled Bears. Zoo Biol..

[86] Carlstead K., Paris S., Brown J.L. (2019). Good keeper-elephant relationships in North American zoos are mutually beneficial to welfare. Appl. Anim. Behav. Sci..

[87] Ward S.J., Melfi V. (2015). Keeper-Animal Interactions: Differences between the Behaviour of Zoo Animals Affect Stockmanship. PLoS ONE.

