# Supplementary material for: Developing a Reliable Welfare Assessment Tool for Captive Hibernatory Bear Species
Source: Animals (Basel). 2021 Oct 29;11(11):3090. doi: 10.3390/ani11113090 (PMC8614556; doi:10.3390/ani11113090)
Supplement: Supplementary file 1 [file animals-11-03090-s001.zip › File_S1_Supplementary_Material.pdf]

Bear welfare assessment guide

Visual assessment

Please note any abnormality e.g. discharges, wounds, damage to tissue, hairloss, etc

	Comments:
Eyes	
Nose	
Teeth	
Skin	
Haircoat	
Claws	
Footpads	
Injuries/wounds	
Skin condition and wounds (please note): 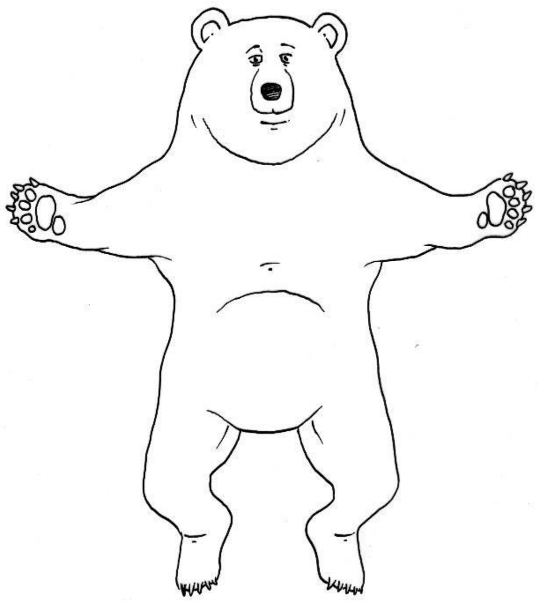	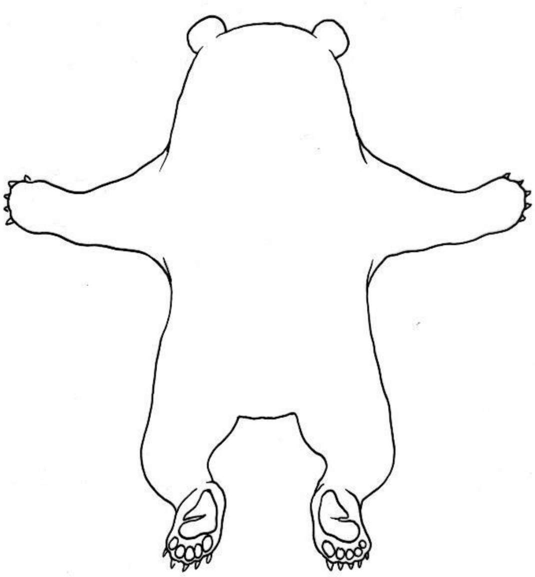

Courtesy of AnimalsAsia.org

Body Condition score

1	2	3	4	5	6	7	8	9
Emaciated. Pelvis and scapulae protruding, ribs visible. A deep hollow will be noted between the pelvis and last rib showing virtually no fat.	Pelvis easily visible, and scapulae still noticeable. Last rib still visible, but ribs have some muscle covering them. The hollow between the pelvis and last rib is obvious.	Pelvis easily visible, ribs no longer visible, having some muscle covering them. The hollow between the pelvis and last rib obvious, but softer.	Slight angle to pelvis, but otherwise body is fully fleshed. The hollow between the pelvis and last rib is small and soft.	Body is fully fleshed out. Some fat is present over pelvis and shoulders. The hollow between the pelvis and last rib is absent.	Body is fully fleshed out. Obvious fat is present over pelvis and shoulders, ribs cannot be seen. The hollow between the pelvis and last rib is absent.	Bear has a rounded or blocky appearance, very well fleshed over all bony areas, obvious fat over rump and shoulders.	Bear has an obvious blocky appearance, very well fleshed over all areas. Obvious fat over rump and shoulders. May have mammary, hindlimb fat causing 'saggy' appearance	Legs appear too short for the body, rolls of fat on neck and lower shoulders. Obvious fat under abdomen

Mobility Assessment (Hindlimb):

**No**		**Description**
1	Good easy Full Stand	Straight up to standing position, no hesitation, hind limbs straight.
2	Full Stand	Slow and stiff but did full stand
3	Half Stand	Managed to stand with front limbs half way up the bars but hind legs straight.
4	Pull up	Sat on ground and pulled up to standing position with front limbs
5	Squatting/Teddy bear	In upright position but either squatting with hindlimbs or sitting in 'teddy-bear' position with hindlimbs forward
6	No stand	Tried to stand but could not.

Gait Assessment (Cincinnati, adapted):

Please tell us what specific activities have become most troublesome to you or your bear

	0 No Problem	1 A little bit difficult	2 Quite difficult	3 very difficult	4 Impossible

	0 No Problem	1 A little bit difficult	2 Quite difficult	3 very difficult	4 Impossible
Walking					
Running					
Jumping/wrestling					
Getting up					
Lying down					
Climbing up (enclosure furnishings, steps etc)					
Descending (enclosure furnishings, steps etc)					
Posturing to urinate or defecate					

Transformed Scale/ 100 = <u>Actual Score x 100</u>
Possible Score
