# Supplementary material for: Developing a Reliable Welfare Assessment Tool for Captive Hibernatory Bear Species
Source: Animals (Basel). 2021 Oct 29;11(11):3090. doi: 10.3390/ani11113090 (PMC8614556; doi:10.3390/ani11113090)

Bear Welfare Assessment Tool:

Training Aid

INTRODUCTION

This is a training aid to help you understand and use the bear welfare assessment tool.

It has been found, through previous research, that assessment tool training is important to increase the effectiveness and accuracy of assessments.

There are currently no tools to assess the welfare of bears. A bear welfare assessment tool was developed last year through expert consensus and through research of bear ecology.

We need to determine if using the tool results in consistent ratings. You know your bears better than anyone and we need your input to further develop the tool.

WHAT IS THE TOOL?

The tool consists of multiple indicators of bear welfare. These include measures of health, behaviour and environment. This tool is designed to be used on multiple bear species, including both temperate and tropical species.

WHAT WILL YOU BE DOING?

You will be using the tool to score each bear by giving them a numerical score/rating. The assessment is expected to take approximately 20 minutes to complete.

We also ask that you provide general information about the bear and your assessment including the bear's name, species, age, sex, life history (if known), the date and time of the assessment, and the length of time required to complete the assessment.

You will need to fill out the Visual Assessment Checklist and the Assessment Tool by hand and then email the research team the scanned documents.

Most of the indicators can be scored quickly by taking observations of the bear and its environment.

It is also important to note that what may be considered 'normal' for an individual may not be normal for the species. Therefore, when performing the assessment, we ask that you follow the scoring system detailed and mark any abnormalities as such, regardless of the 'normal' health and behaviour of the individual bear being assessed.

Also, it would be helpful to the research team if you could perform the assessment three times across nine days. However, if this is not possible then performing a single assessment is sufficient.

THE ASSESSMENT

Physical Health

1. Weight

Sex	American Black bear	Brown bear	Polar bear	Panda	Sun bear	Andean bear	Sloth bear	Asiatic black bear
Male (kg)	60 to 300	135 to 770	400 to 600	85 to 160	27 to 65	100 to 155	80 to 140	100 to 200
Female (kg)	40 to 150	95 to 365	200 to 300	70 to 125	27 to 65	64 to 82	55 to 95	50 to 125

If the bear's weight falls into the normal weight range for the species (or sub-species), then it is given a score of 3. If the bear's weight is outside of the normal weight range (underweight or overweight), then it is given a score of 1.

This is a measure that can be determined by weighing the bear, or if this is not possible, estimated visually.

2. Body Condition Score (BCS)

On the Visual Assessment Checklist, which is part of the assessment, there is a table that lays out a 9-point scale detailing how to assess BCS (see table below). This can be assessed through a visual inspection of the bear.

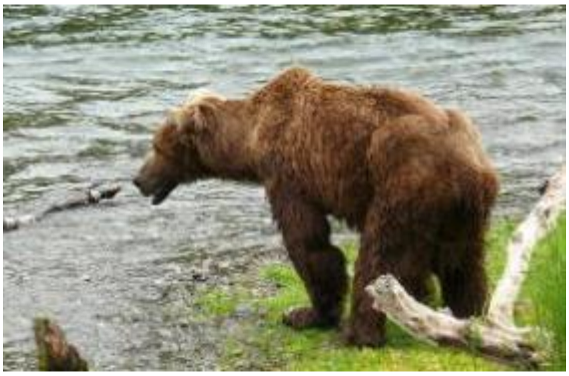

BCS Score 1 (underweight)

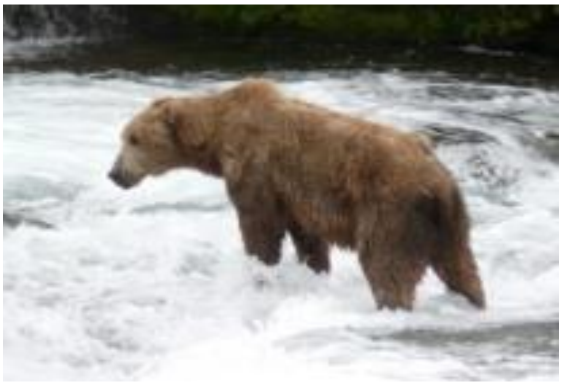

BCS Score 2 (underweight)

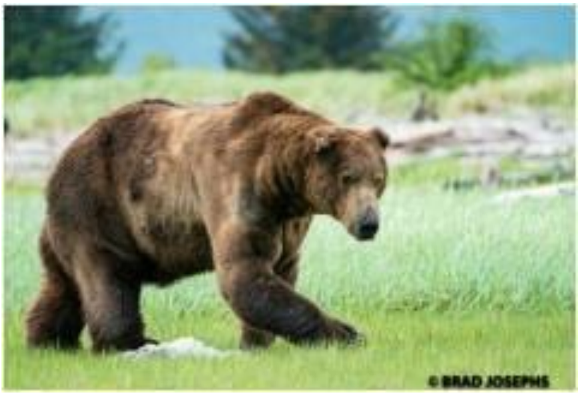

BCS Score 3 (underweight)

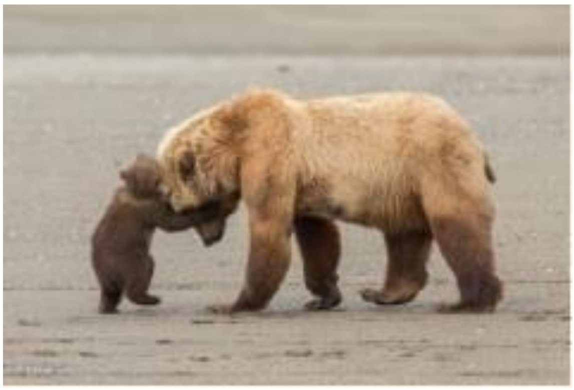

BCS Score 4 (normal weight)

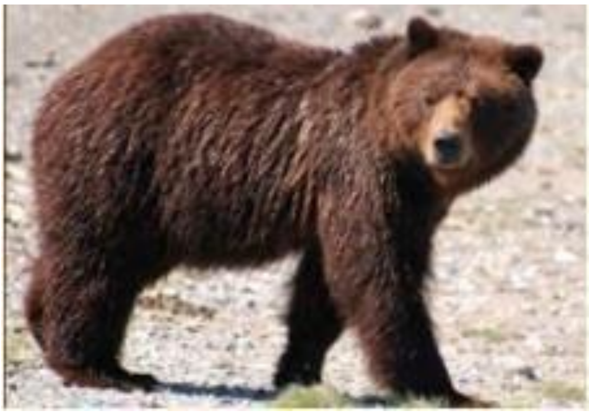

BCS Score 5 (normal weight)

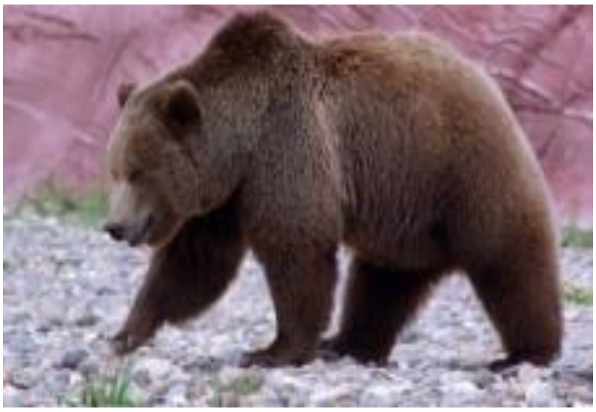

BCS Score 6 (normal weight)

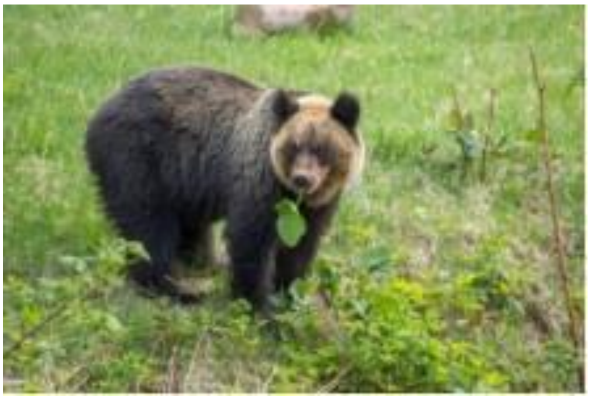

BCS Score 7 (overweight)

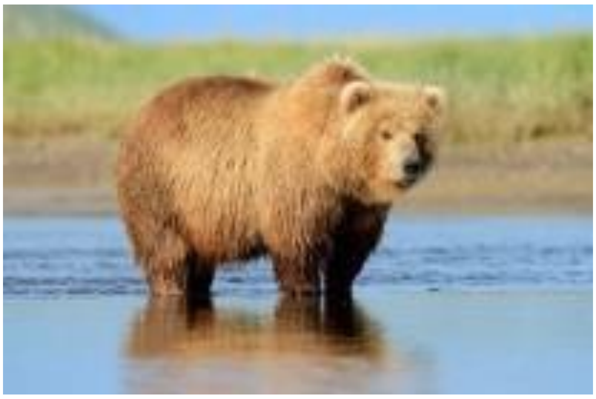

BCS Score 8 (overweight)

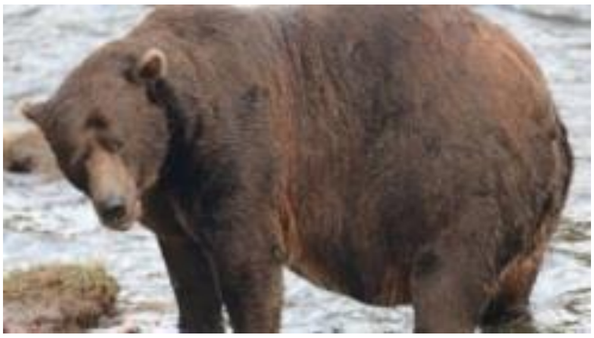

BCS Score 9 (overweight)

Bears that are rated as either 1, 2, 3, 7, 8 or 9 are given a welfare score of 1. Bears that are rated as either 4, 5 or 6 are given a welfare score of 3.

Body Condition Score Table

1	2	3	4	5	6	7	8	9
Emaciated. Pelvis and scapulae protruding, ribs visible. A deep hollow will be noted between the pelvis and last rib showing virtually no fat.	Pelvis easily visible, and scapulae still noticeable. Last rib still visible, but ribs have some muscle covering them. The hollow between the pelvis and last rib is obvious.	Pelvis easily visible, ribs no longer visible, having some muscle covering them. The hollow between the pelvis and last rib obvious, but softer.	Slight angle to pelvis, but otherwise body is fully fleshed. The hollow between the pelvis and last rib is small and soft.	Body is fully fleshed out. Some fat is present over pelvis and shoulders. The hollow between the pelvis and last rib is absent.	Body is fully fleshed out. Obvious fat is present over pelvis and shoulders, ribs cannot be seen. The hollow between the pelvis and last rib is absent.	Bear has a rounded or blocky appearance, very well fleshed over all bony areas, obvious fat over rump and shoulders.	Bear has an obvious blocky appearance, very well fleshed over all areas. Obvious fat over rump and shoulders. May have mammary, hindlimb fat causing 'saggy' appearance	Legs appear too short for the body, rolls of fat on neck and lower shoulders. Obvious fat under abdomen

- <u>Visual Assessment</u>

The visual assessment involves a visual inspection of the bear's outward appearance. You are looking for indications of discharge, wounds, tissue damage, bleeding and other signs of illness or injury. If any abnormalities are seen or there are any deviations from what is considered normal health and appearance for the species, the bear is given a welfare score of 1. If there are no abnormalities and the area being examined appears healthy, then the bear is given a welfare score of 3.

There is a Visual Assessment Checklist sheet that accompanies the welfare assessment. On this checklist, we ask that you comment about what kind of abnormalities that you observe and that you mark these on the bear diagram.

3. Eyes;

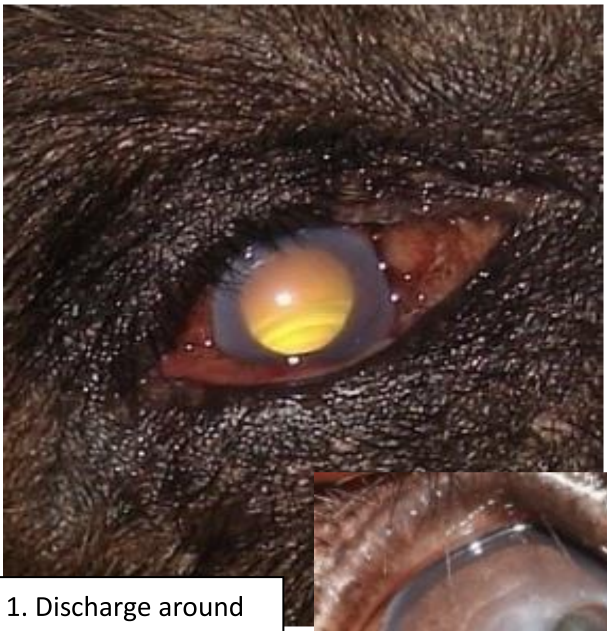

1. Discharge around the eye. Welfare score of 1.

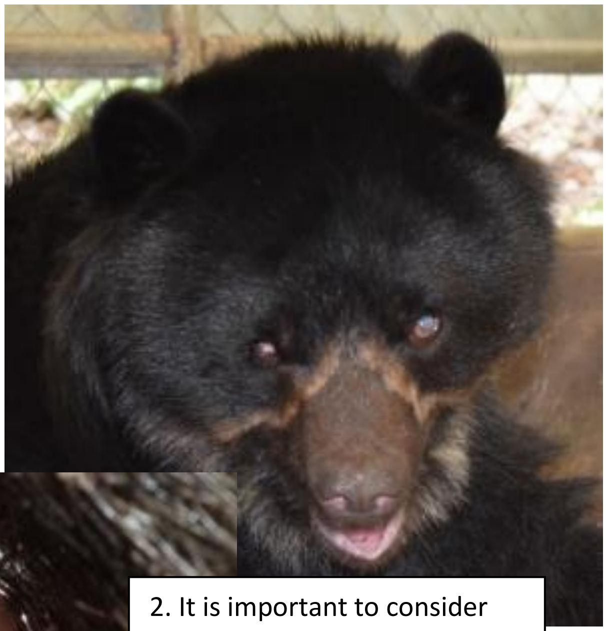

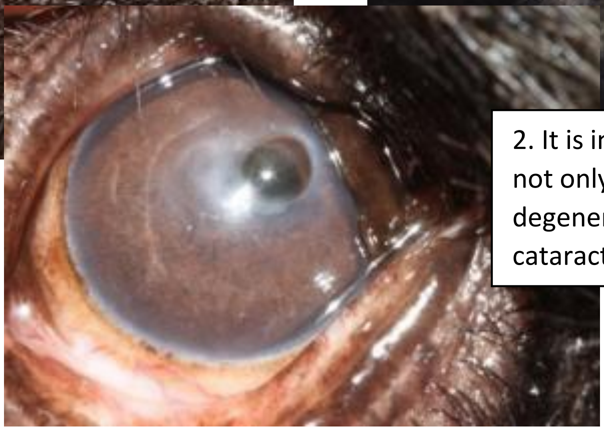

2. It is important to consider not only injuries but degenerative disorders such as cataracts.

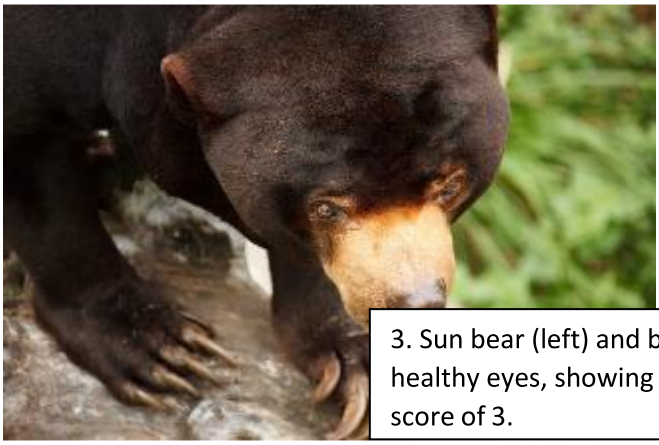

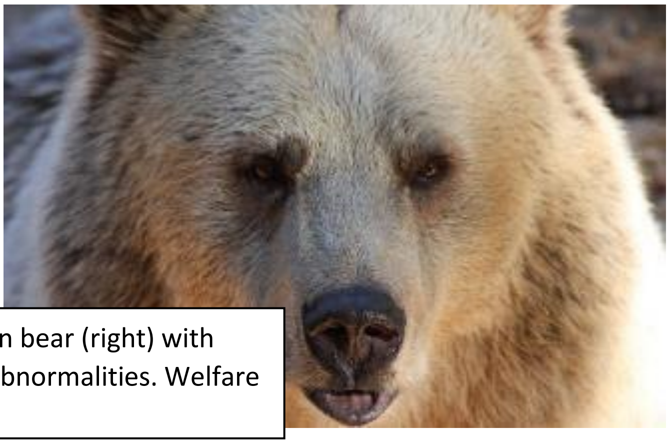

3. Sun bear (left) and brown bear (right) with healthy eyes, showing no abnormalities. Welfare score of 3.

4. Nose;

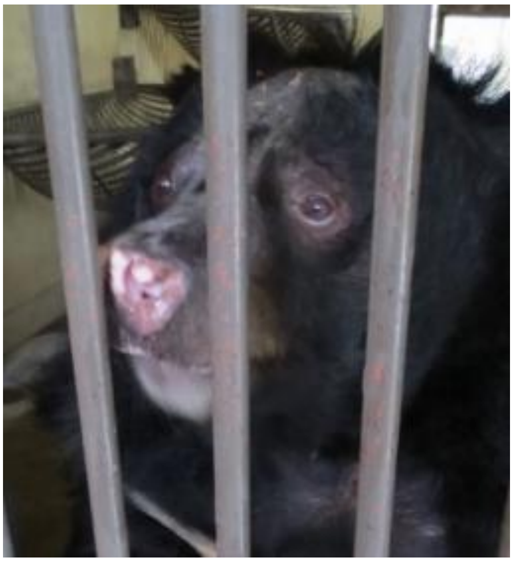

1. Nose Injury. Welfare score of 1.

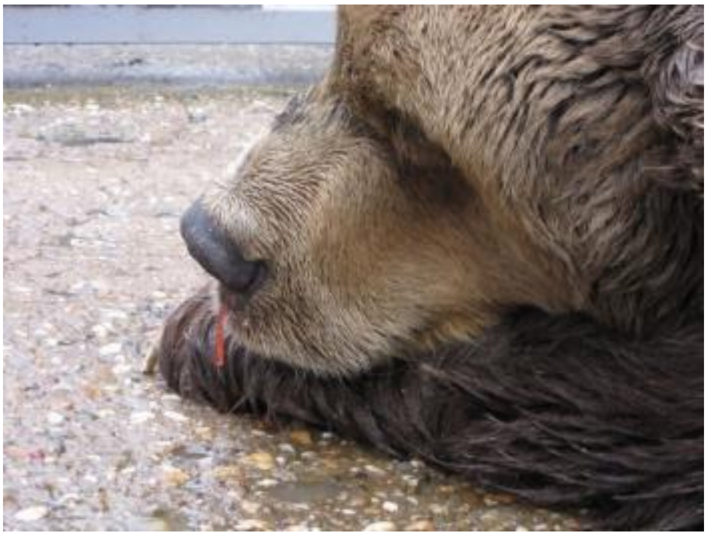

2. Discharge or bleeding. Welfare score of 1.

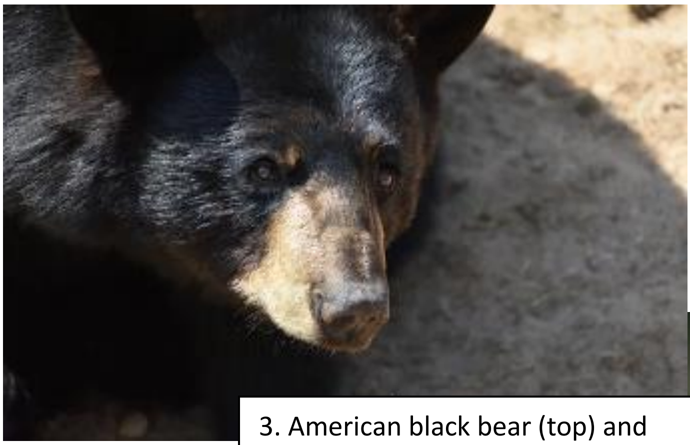

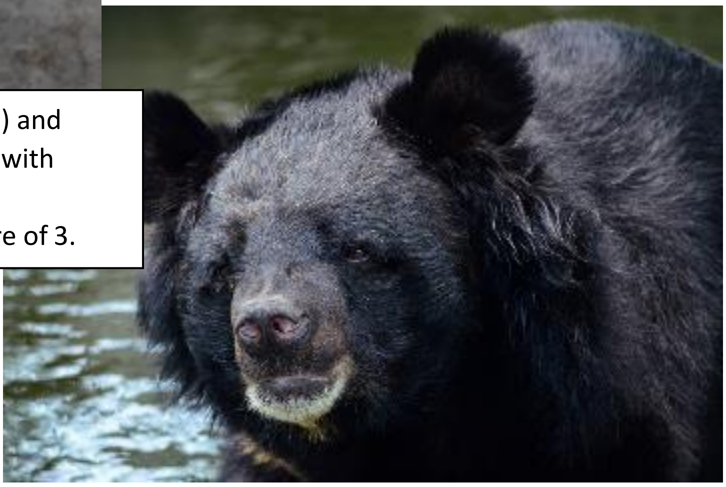

3. American black bear (top) and Asiatic black bear (bottom) with healthy noses, showing no abnormalities. Welfare score of 3.

5. Teeth;

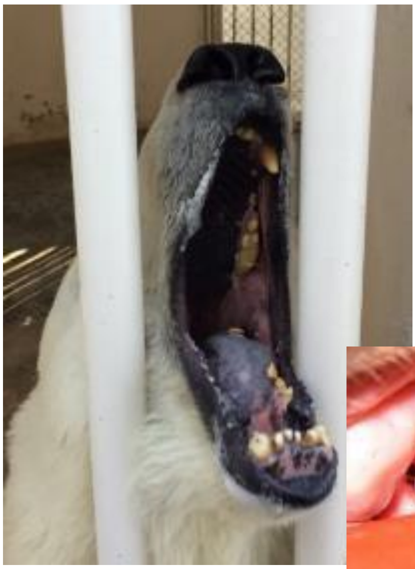

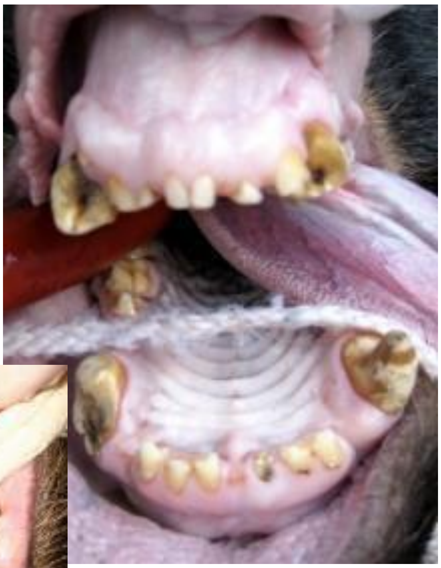

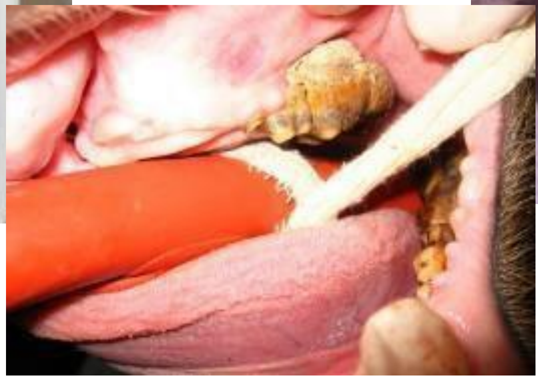

1. Examples of broken and rotting teeth. Welfare score of 1.

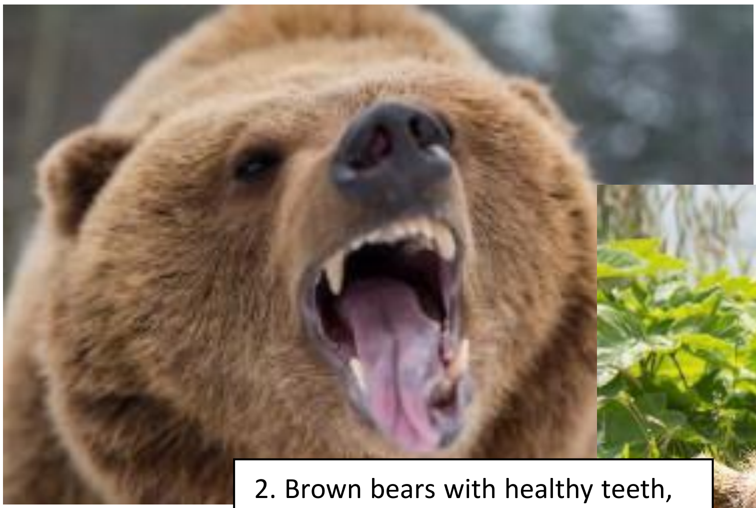

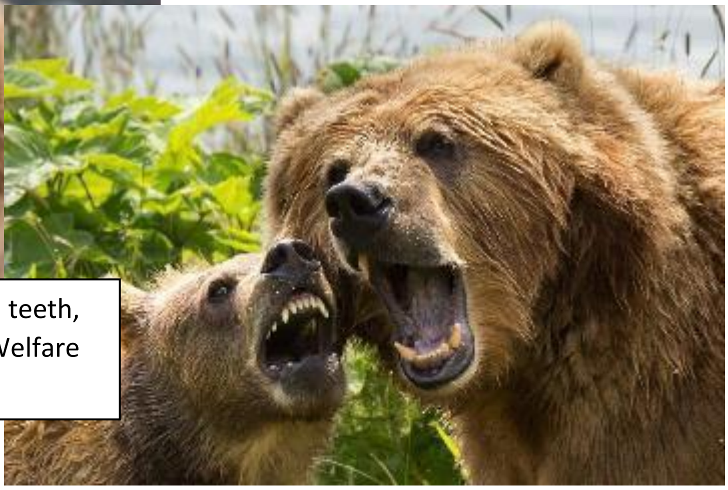

2. Brown bears with healthy teeth, showing no abnormalities. Welfare score of 3.

6. Skin (including signs of infections, allergies, hair-loss on rump from repeated sitting);

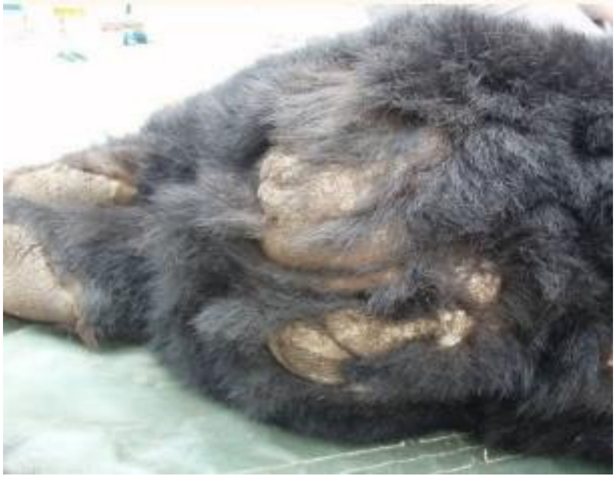

1. Skin issues on rump. Welfare score of 1.

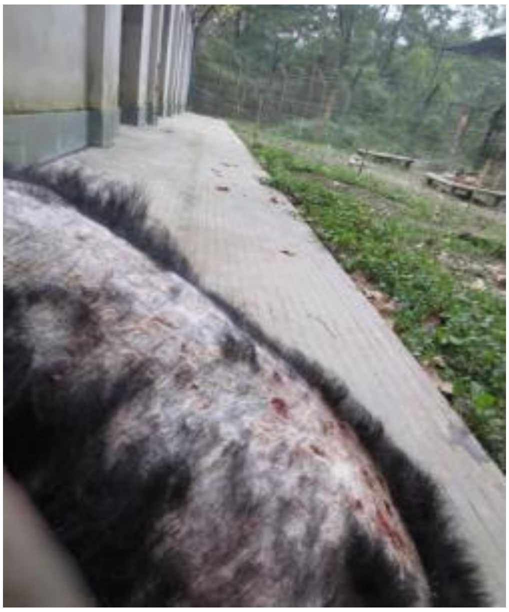

2. Skin issues on body. Welfare score of 1.

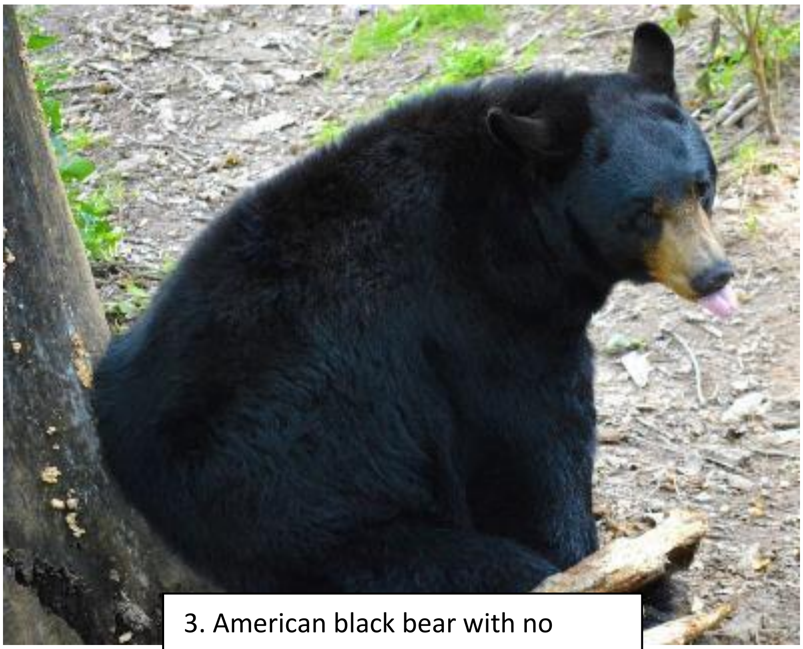

3. American black bear with no apparent skin problems. Welfare score of 3.

7. Haircoat (including signs of matting, hair-loss, colouration change, reduced grooming behaviour, unkempt);

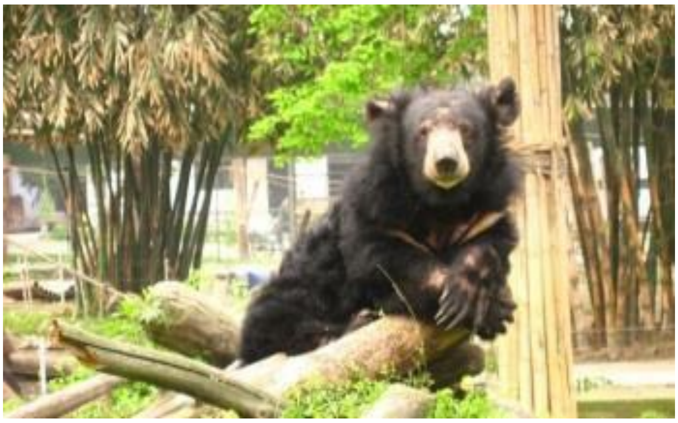

1. Asiatic black bear with unkempt coat with hair-loss/thinning around paws. Welfare score of 1.

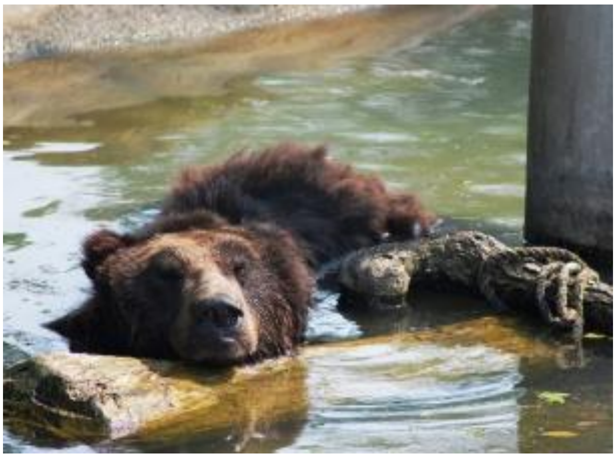

2. Brown bear with unkempt coat. Welfare score of 1.

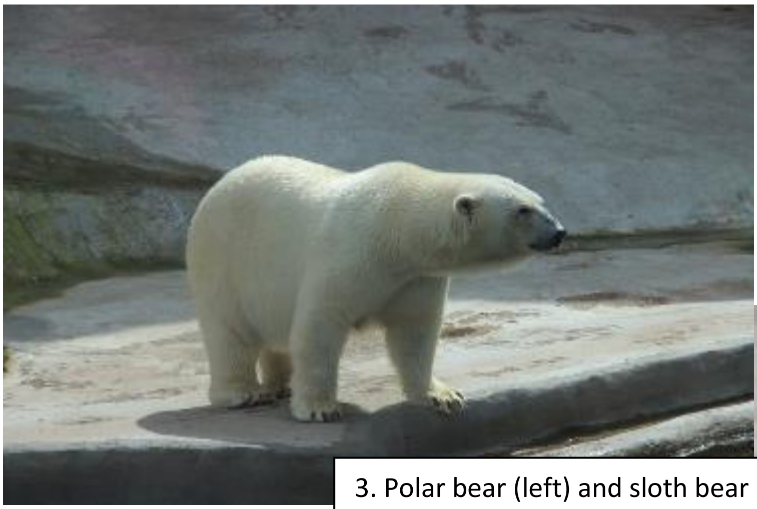

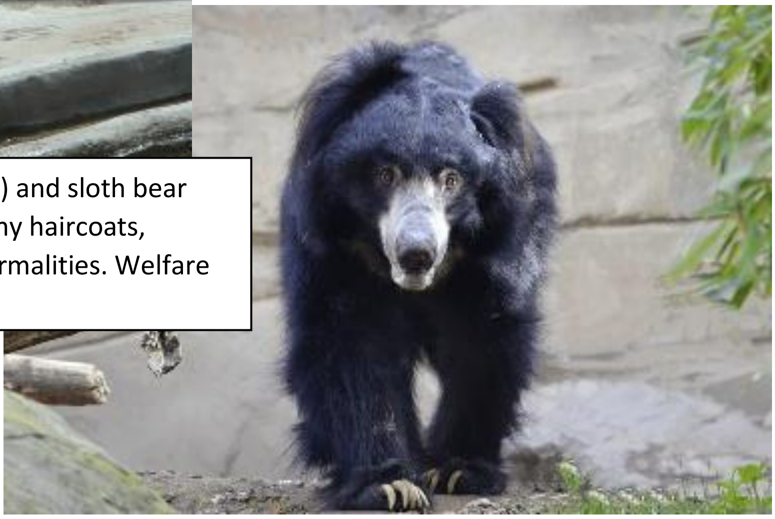

3. Polar bear (left) and sloth bear (right) with healthy haircoats, showing no abnormalities. Welfare score of 3.

8. Claws (including overgrown, broken, infected, amputated);

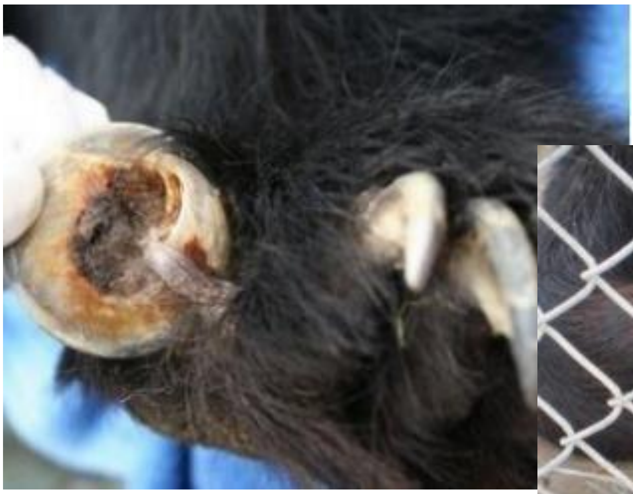

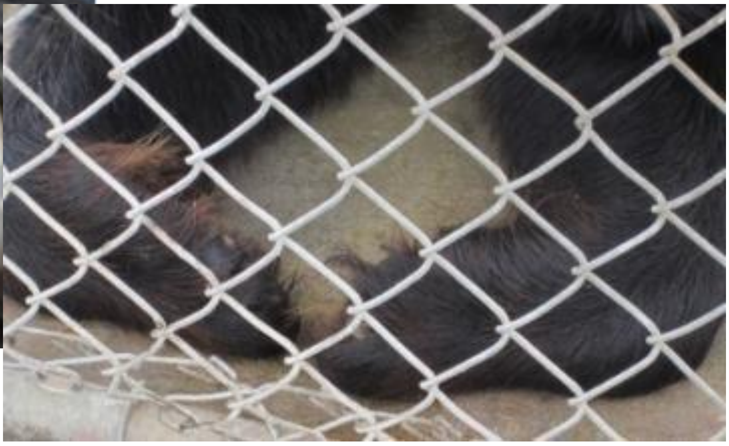

1. Broken or infected claws are given a welfare score of 1.

2. Claws removed. Welfare score of 1.

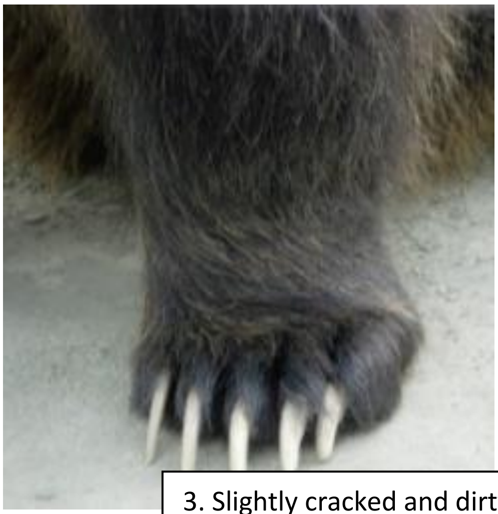

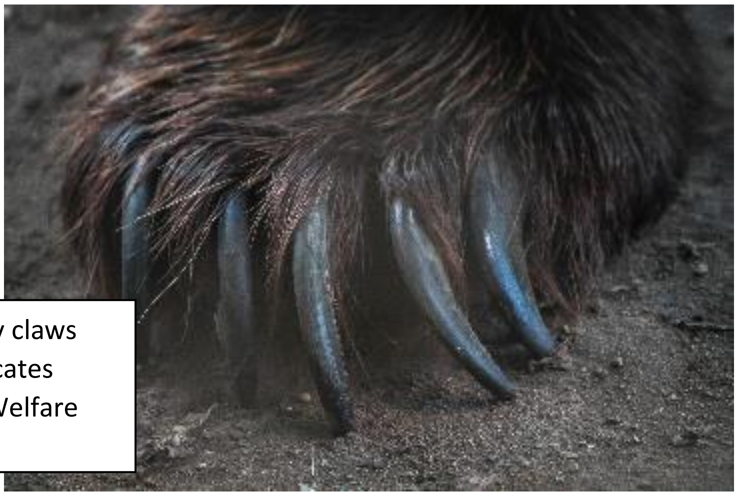

3. Slightly cracked and dirty claws are a good sign as this indicates activities such as digging. Welfare score of 3.

9. Footpads (including signs of thickening, dry, cracked, ulcerated);

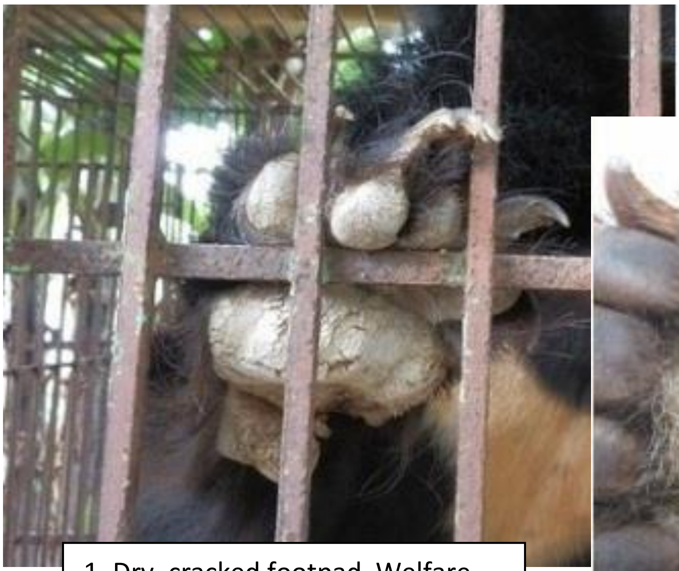

1. Dry, cracked footpad. Welfare score of 1.

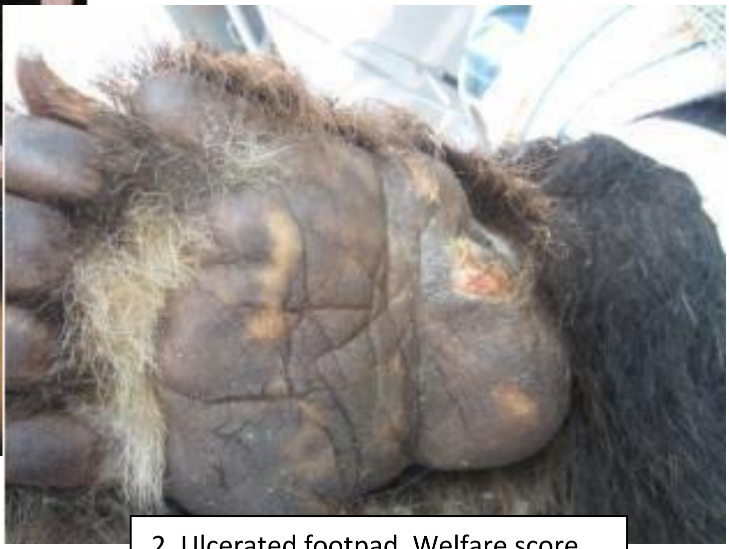

2. Ulcerated footpad. Welfare score of 1.

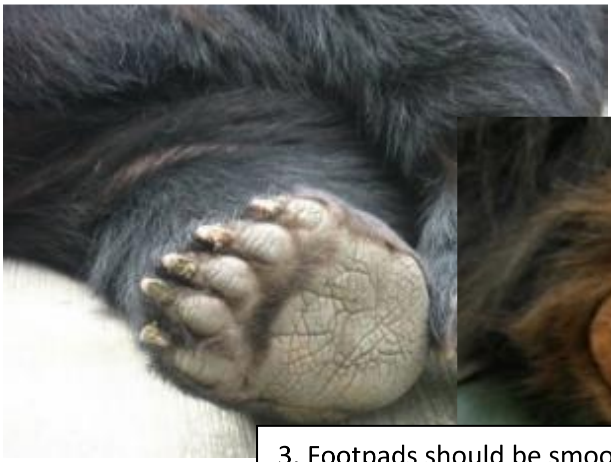

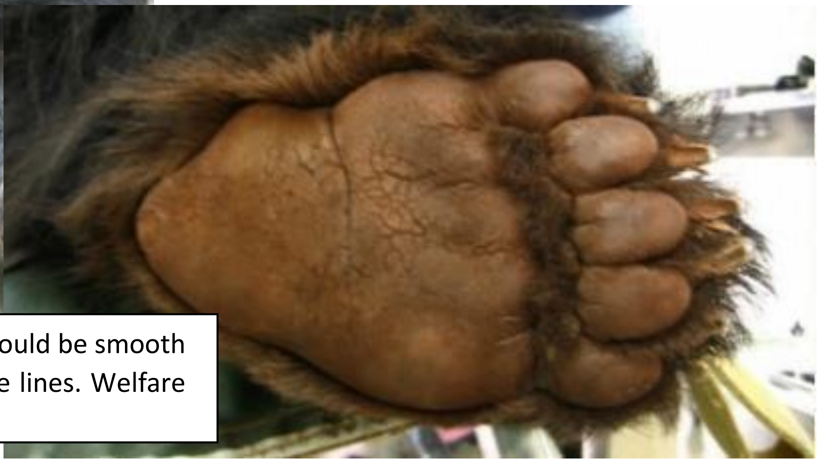

3. Footpads should be smooth with only some lines. Welfare score of 3.

10. Injuries/Wounds;

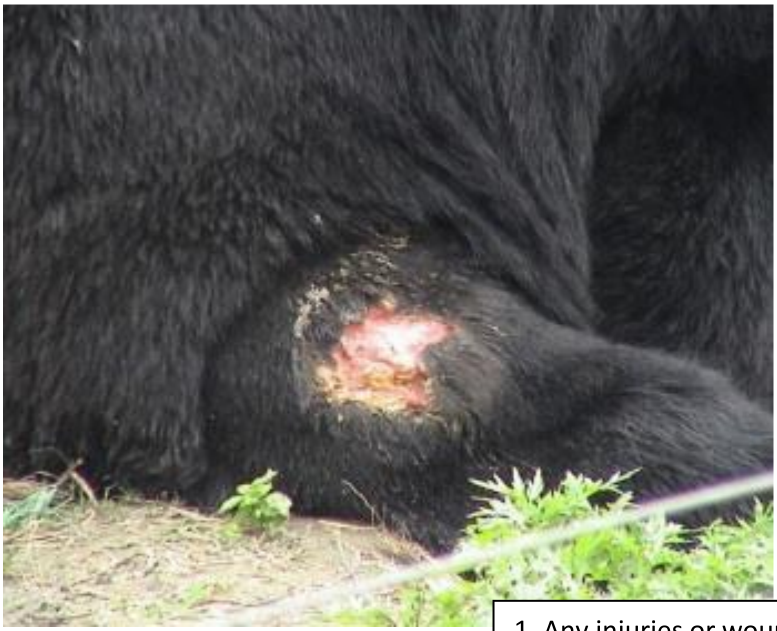

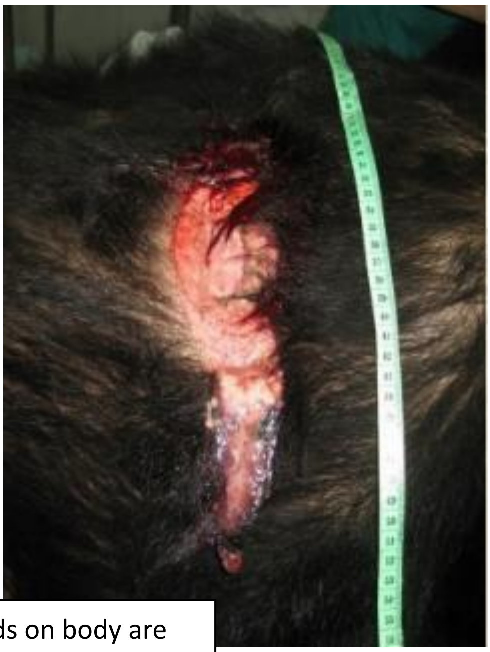

1. Any injuries or wounds on body are given a welfare score of 1.

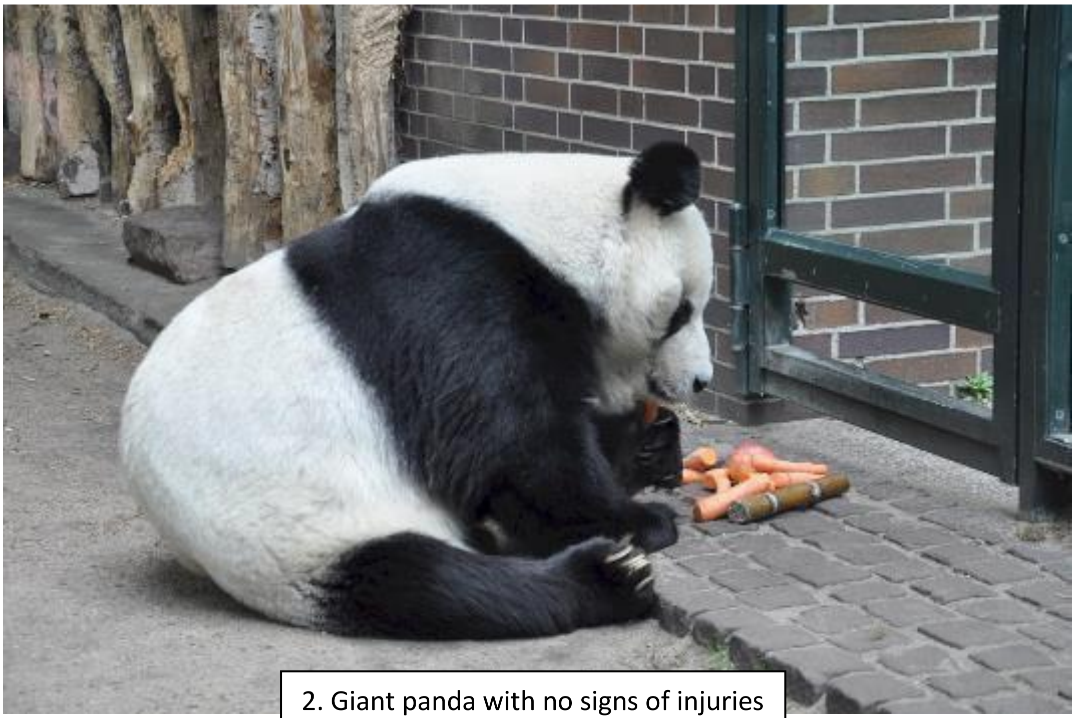

2. Giant panda with no signs of injuries or wounds. Welfare score of 3.

11. Mobility Hindlimb Assessment

There is a 7-point scale detailed on the accompanying Welfare Assessment Checklist. A grade of 1 is given a welfare score of 3. A grade of 2 is given a welfare score of 2. A grade of 3 or 4 is given a welfare score of 1. Finally, a grade of 5 or 6 is given a welfare score of 0 (see table below). Bear hindlimb mobility should be assessed by encouraging your bear to stand fully on their hindlimbs in a safe, enclosed area.

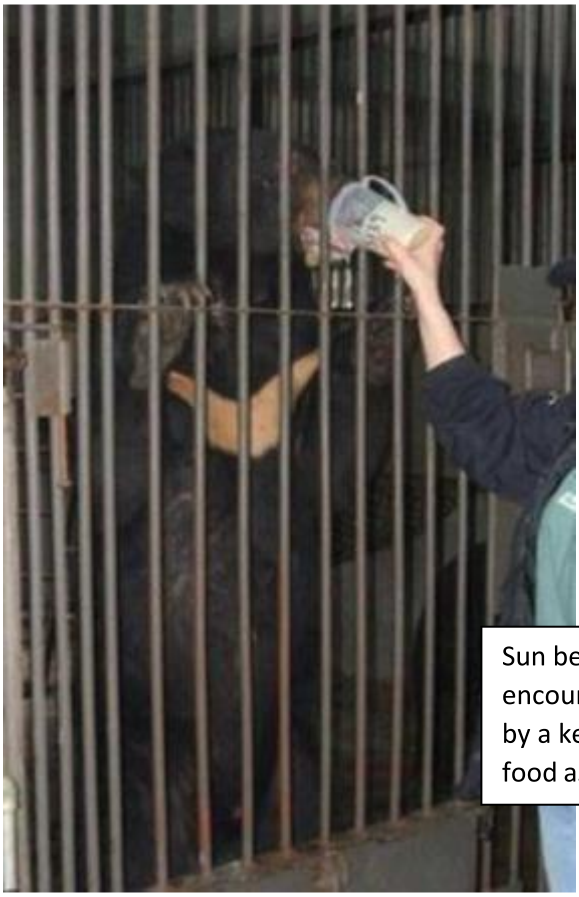

Sun bear being encouraged to stand by a keeper using food as a reward.

Mobility Assessment Hindlimb Table:

No		Description
1	Good easy Full Stand	Straight up to standing position, no hesitation, hind limbs straight.
2	Full Stand	Slow and stiff but did full stand
3	Half Stand	Managed to stand with front limbs half-way up the bars but hind legs straight.
4	Pull up	Sat on ground and pulled up to standing position with front limbs
5	Squatting/Teddy bear	In upright position but either squatting with hindlimbs or sitting in 'teddy-bear' position with hindlimbs forward
6	No stand	Tried to stand but could not.

Examples for Mobility Hindlimb Assessment;

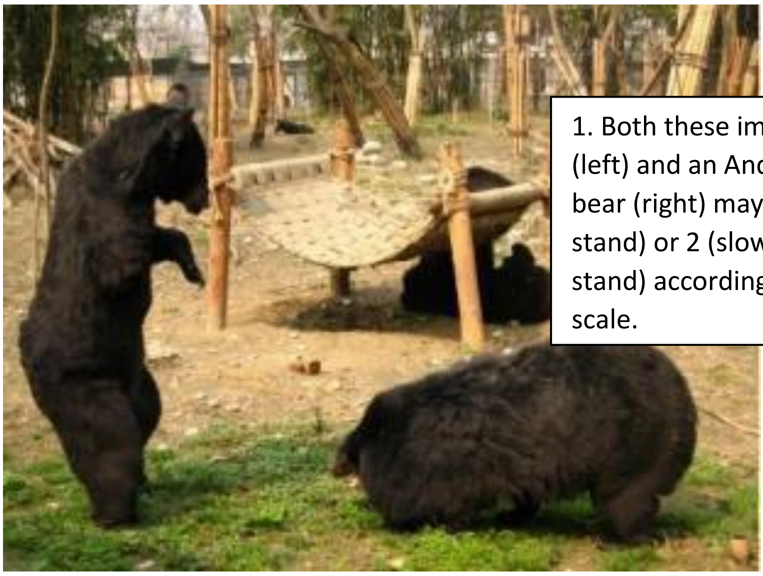

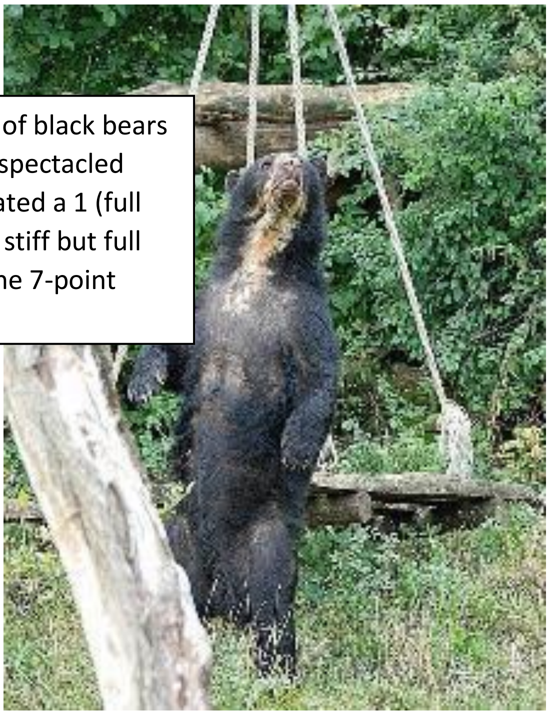

1. Both these images of black bears (left) and an Andean spectacled bear (right) may be rated a 1 (full stand) or 2 (slow and stiff but full stand) according to the 7-point scale.

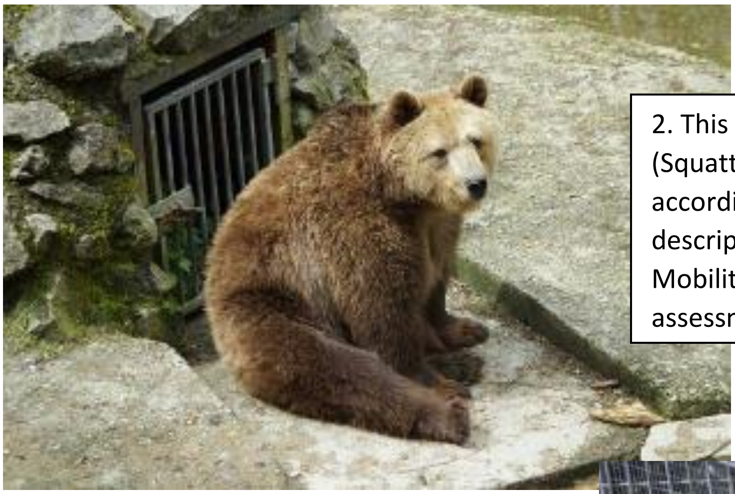

2. This would be rated a 5 (Squatting/Teddy bear) according to the description of the Mobility Hindlimb assessment.

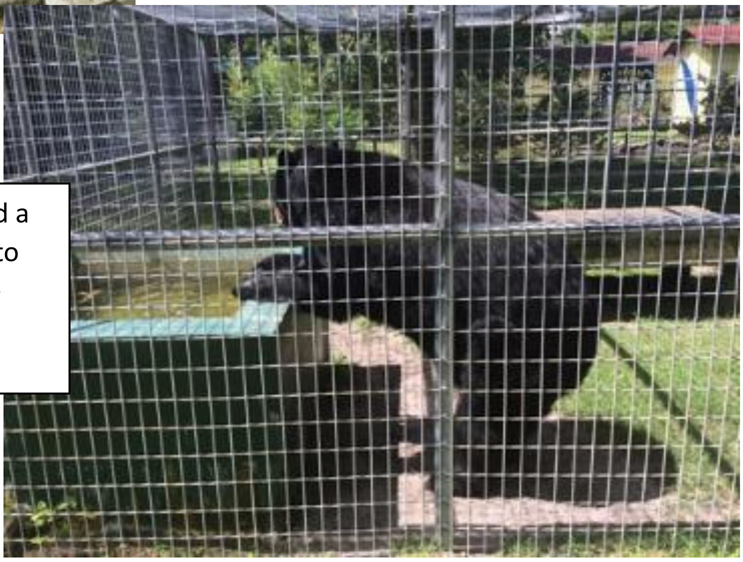

3. This would be rated a 4 (Pull up) according to the description of the Mobility Hindlimb assessment.

12. Gait Assessment (Cincinnati)

This type of gait assessment has already been validated in dogs. There are two parts to this assessment and it can be completed in any order.

The first is a table (Table 1) asking you to list specific activities that your bear enjoys/is motivated to do, but may have become difficult for your bear to perform e.g. climbing, nesting, back-scratching wrestling etc. Once listed, we then ask you to rate these activities on a scale from 0 (No problem) to 4 (Impossible).

The second part is another table (Table 2) which lists common movements including walking, running, lying down etc. Again, you are asked to rate these activities on the same scale.

Table 1.

	0 No Problem	1 A little bit difficult	2 Quite difficult	3 very difficult	4 Impossible

Table 2.

	0 No Problem	1 A little bit difficult	2 Quite difficult	3 very difficult	4 Impossible
Walking					
Running					
Jumping/wrestling					
Getting up					
Lying down					
Climbing up (enclosure furnishings, steps etc)					
Descending (enclosure furnishings, steps etc)					
Posturing to urinate or defecate					

The final thing to do here is to calculate the overall score for the gait assessment. This involves the following equation;

$$\text{Overall Score} = \frac{\text{Actual Score x 100}}{\text{Possible Score}}$$

The Overall Score is the scores from both tables added together, multiply this by 100, then divide by the possible score.

The Possible Score is the number of activities listed and multiplied by 4.

The overall score for the gait assessment is then given a welfare rating. A gait assessment score of 0-10 is given a welfare score of 3. A gait assessment score of 11-40 is given a welfare score of 2. A gait assessment score of 41-60 is given a welfare score of 1. Finally, a gait assessment score of 61 or more is given a welfare score of 0.

Examples of activities for Gait Assessment;

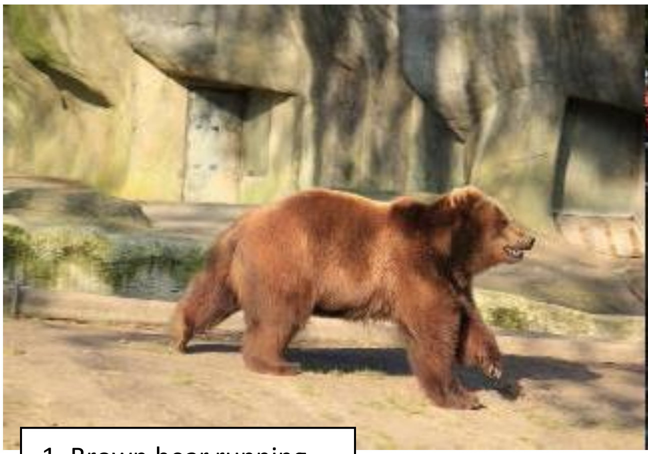

1. Brown bear running.

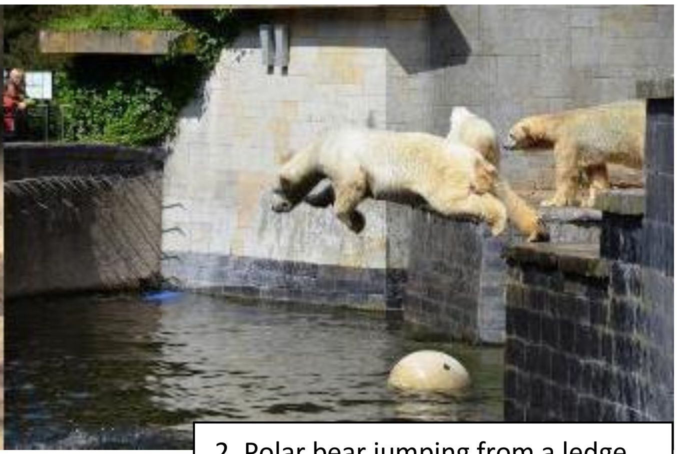

2. Polar bear jumping from a ledge into water.

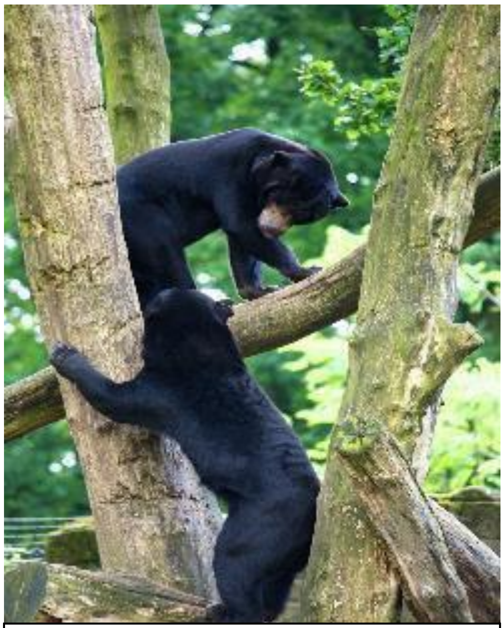

3. Sun bears climbing up enclosure furnishings.

Behaviour

In relation to the observation of behaviours, if a behaviour is observed daily then it scores a 3. If it is observed 2 or 3 times within the last week it scores a 2. If it is observed less than twice within the last week then it scores a 1. Finally, if it is not observed at all, then it scores a 0.

You may not observe certain behaviours at the exact time that you are performing the assessment. In this case, we ask that you to take into consideration how many times you have seen the bear perform this behaviour within the last week from the time that you perform the assessment. For instance, you have observed the bear playing with objects daily for the last seven days but when you are performing the assessment the bear is not playing. You would still rate the bear a welfare score of 3 for object play since you have observed them performing this behaviour regularly.

13. Foraging

Definition: Bear searches for food throughout the environment.

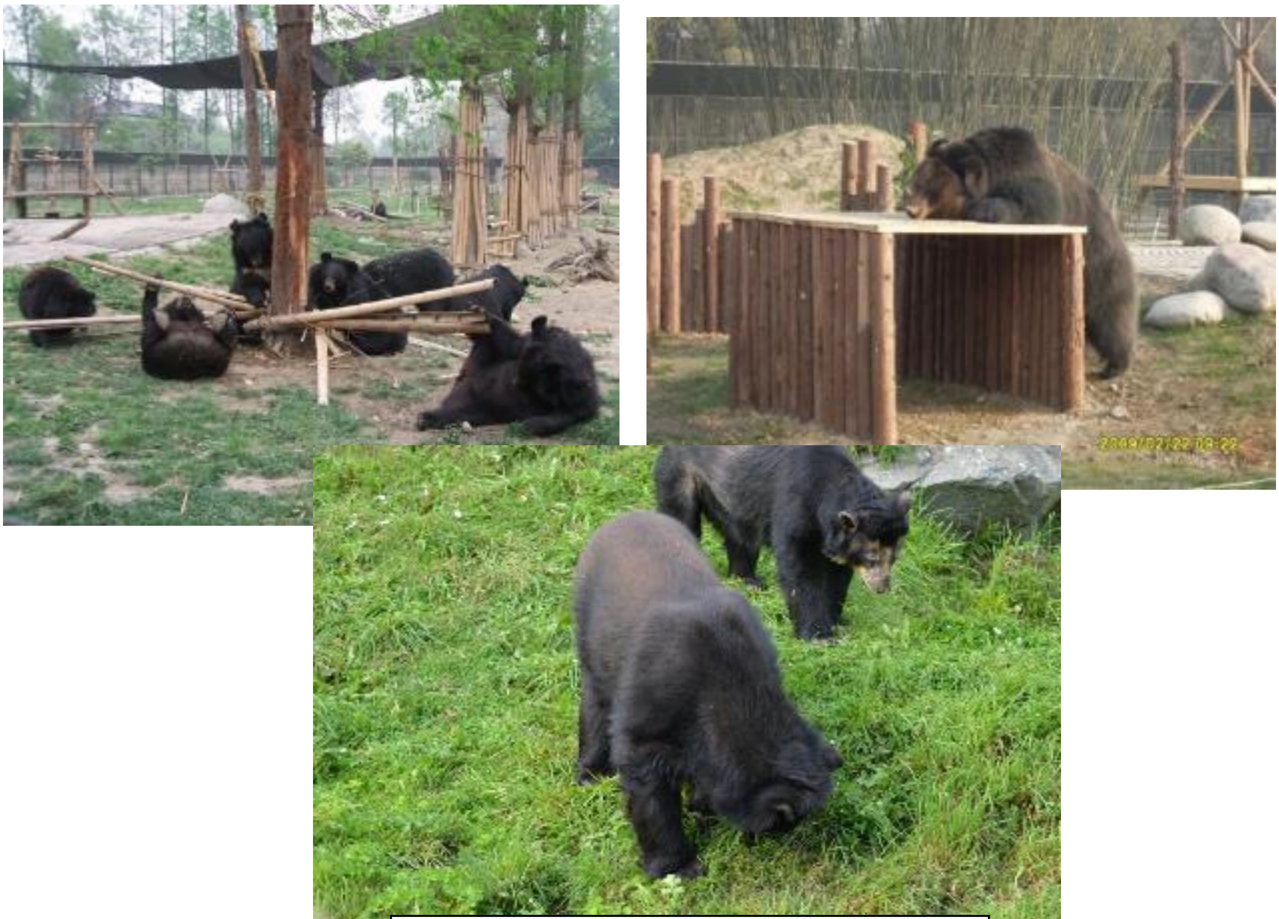

Black bears (top left), brown bear (top right) and Andean spectacled bears (bottom centre) foraging in their environments.

14. Climbing

Definition: Bear uses structures or trees within the enclosure to climb off the ground.

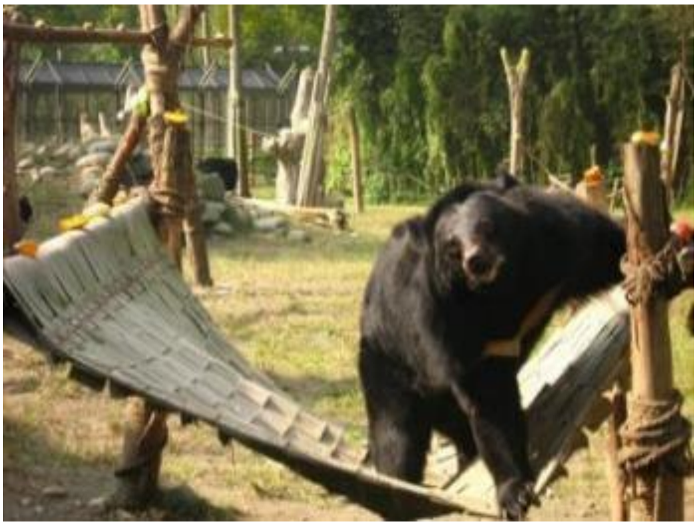

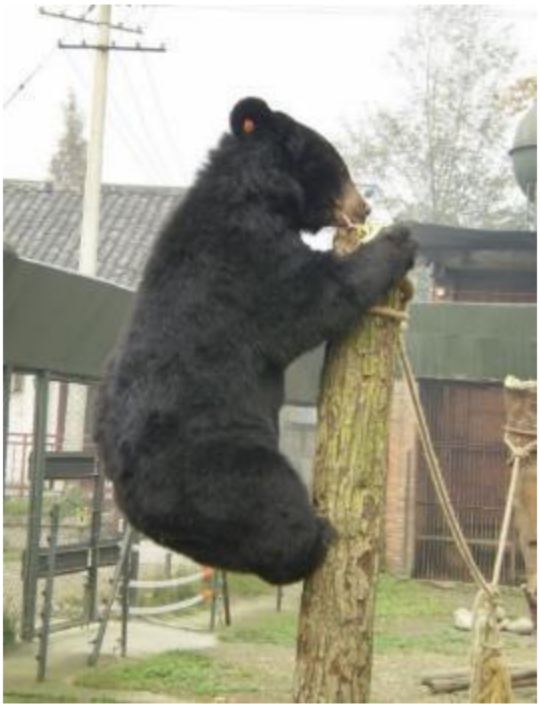

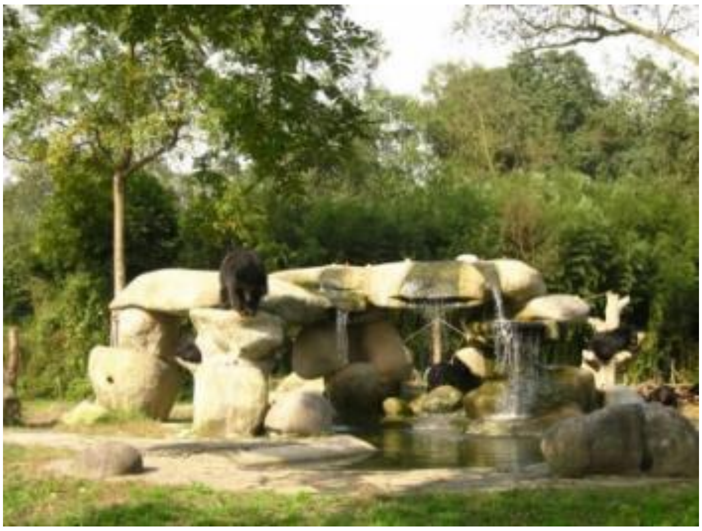

Asiatic black bears (top and bottom left) and American black bear climbing various structures in their environments.

15. Social Play

Definition: Bear interacts positively with other bears, including wrestling/swatting/interacting with soft open mouth.

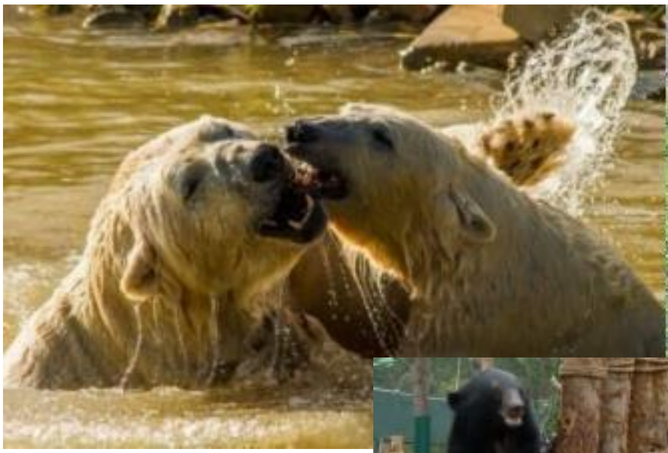
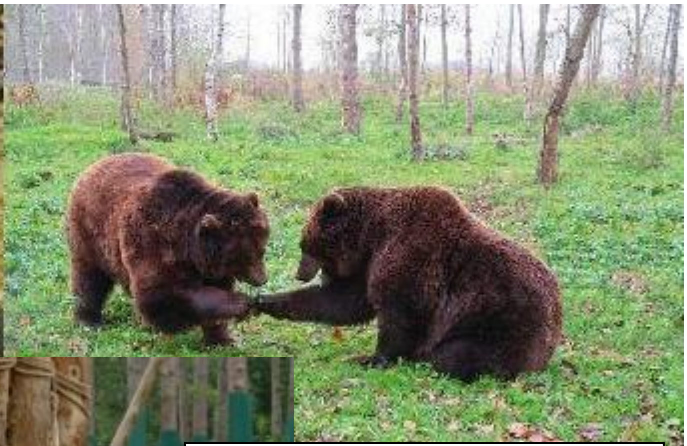
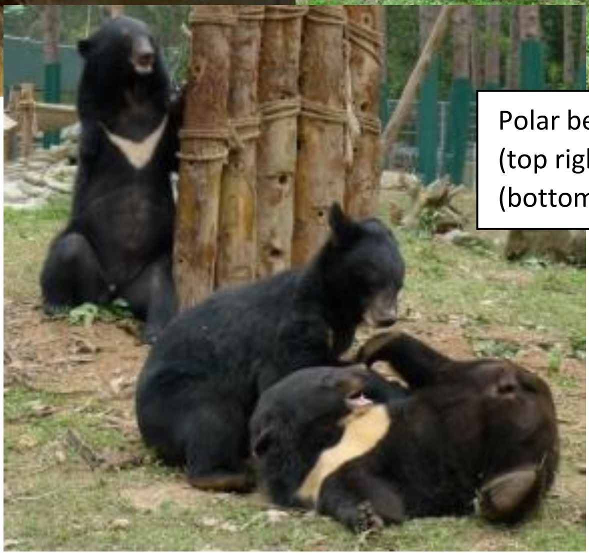

Polar bears (top left), brown bears (top right) and Asiatic black bears (bottom centre) playing together.

Play behaviour includes signs such as puckered lips, relaxed face, ears partially flattened to crescent, wrestling, jaw gaping, play-biting, play-swatting, and lunging. There are no vocalisations associated with play.

16. Object Play

Definition: Bear interacts positively with and non-repetitively with an object.

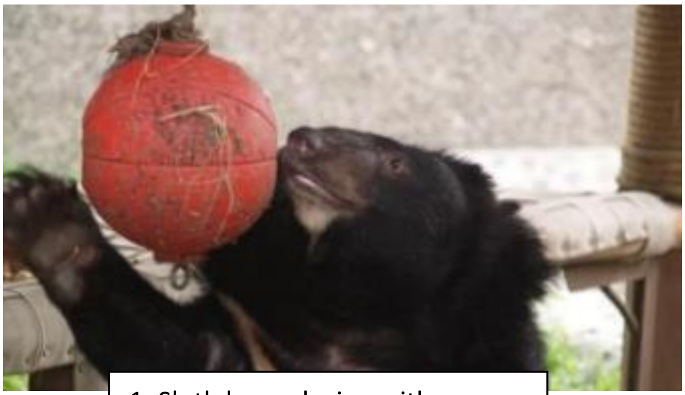

1. Sloth bear playing with a hanging ball.

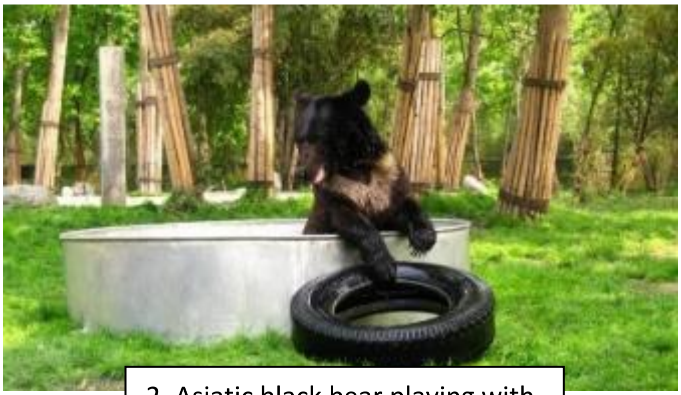

2. Asiatic black bear playing with a tyre.

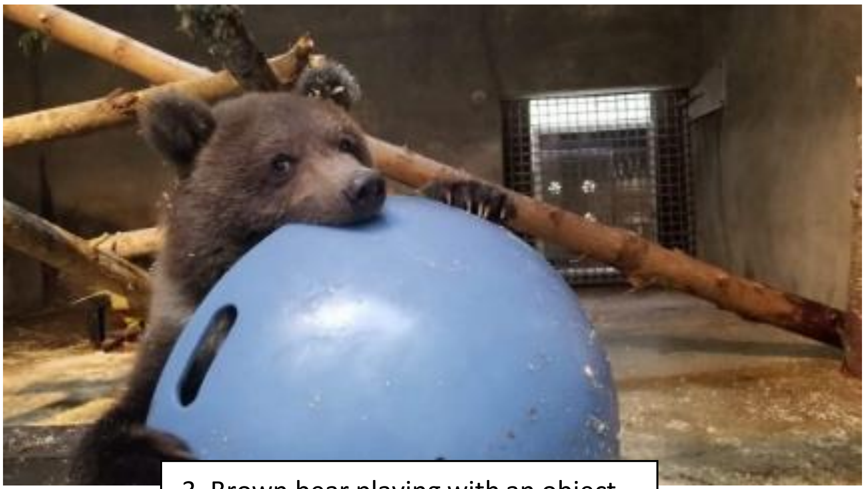

3. Brown bear playing with an object.

17. Nest Building

Definition: Bear gathers provided materials together into a pile to rest on during the day.

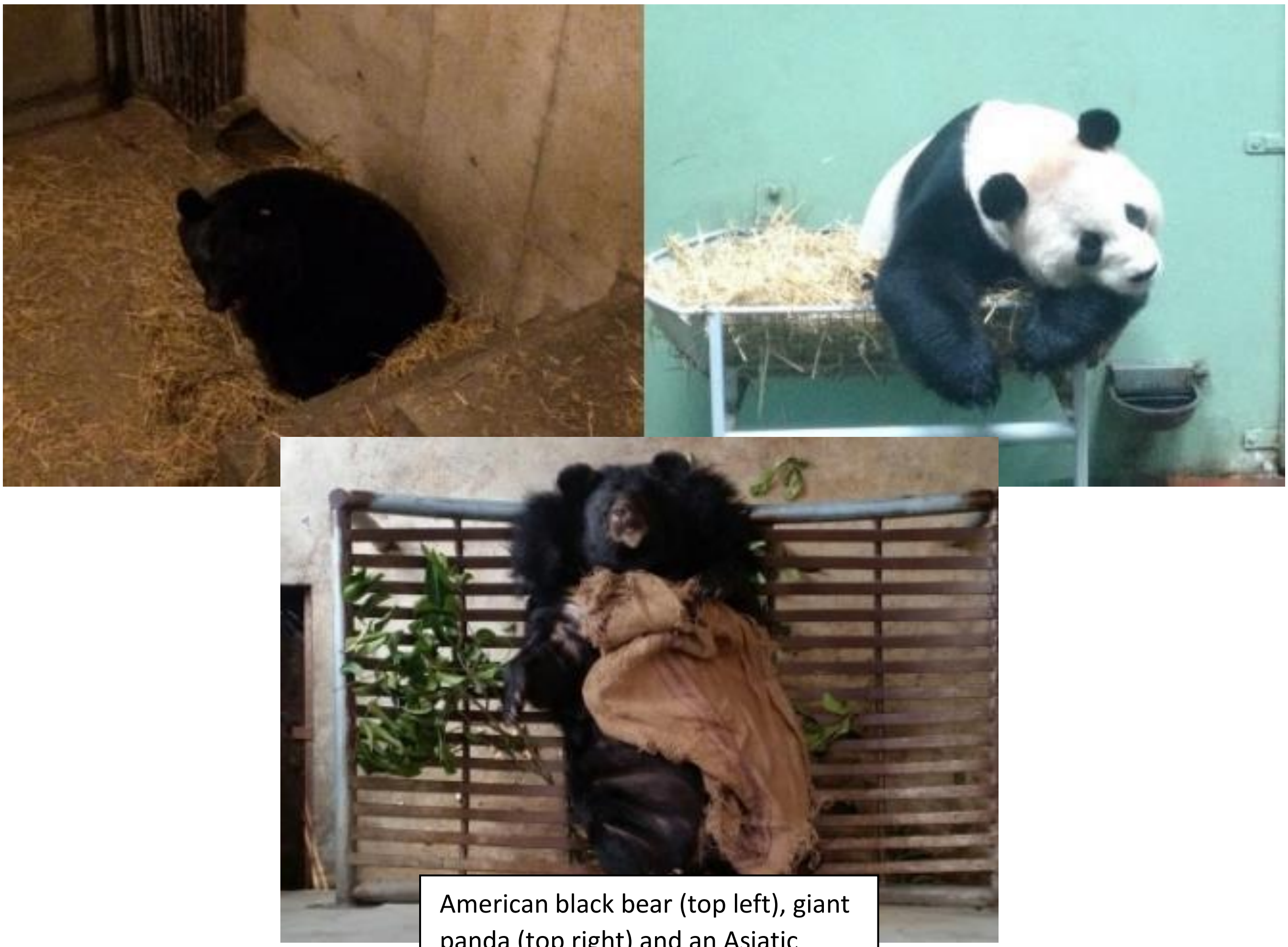

American black bear (top left), giant panda (top right) and an Asiatic black bear using a variety of gathered substrates and materials to create day nests.

Behaviour

Certain behaviours can also be a sign of negative welfare in bears. Aggression and abnormal behaviour/stereotypy are examples of these. For the welfare assessment if these behaviours are observed daily than the bear scores a 0. If they are observed 2 or 3 times per week then the bear scores a 1. If they are observed less than twice a week the bear scores a 2. Finally, if they are not observed at all then the bear scores a 3.

18. Aggression

Definition: Bear displays aggressive behaviour including growling, roaring, charging, or swatting towards another bear or a human. There may also be vocalisations.

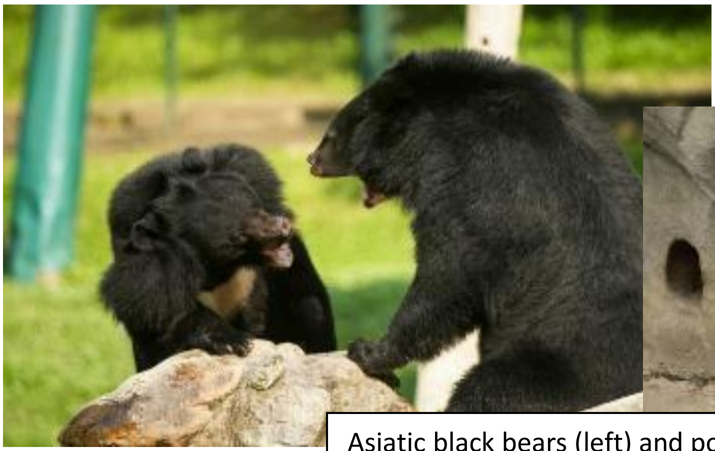

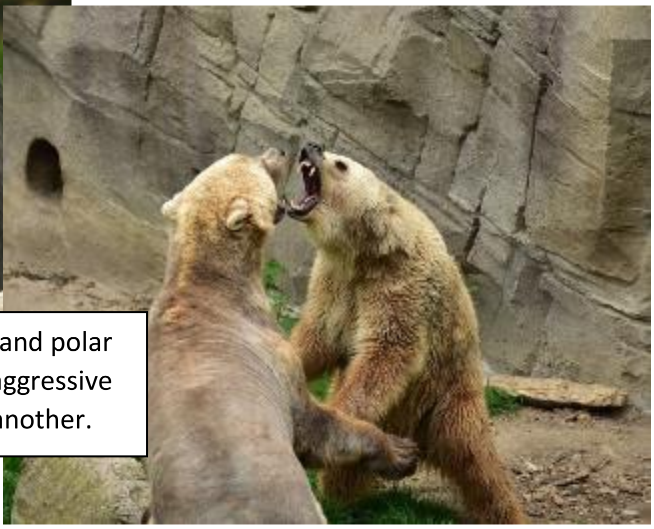

Asiatic black bears (left) and polar bears (right) displaying aggressive behaviour towards one another.

19. Abnormal Behaviour/ Stereotypy

Definition: Bear displays repetitive head-swaying, pacing, rocking, pouncing or object rolling/ placing behaviour.

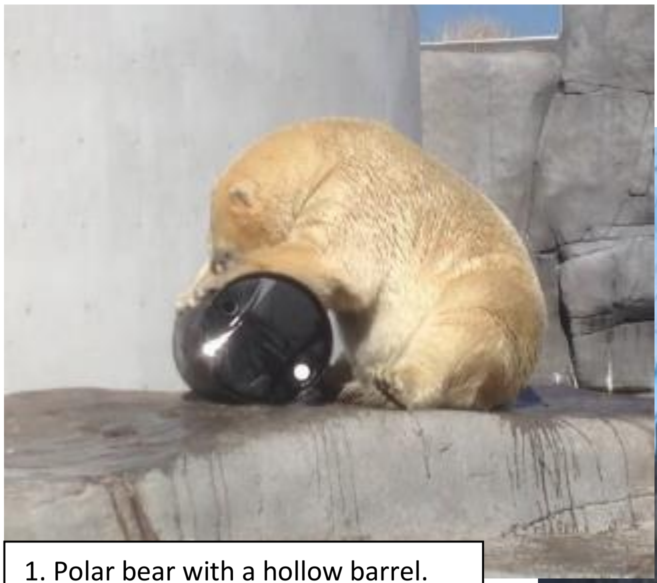

1. Polar bear with a hollow barrel. An example of a stereotypy may be rolling the object back and forth repetitively.

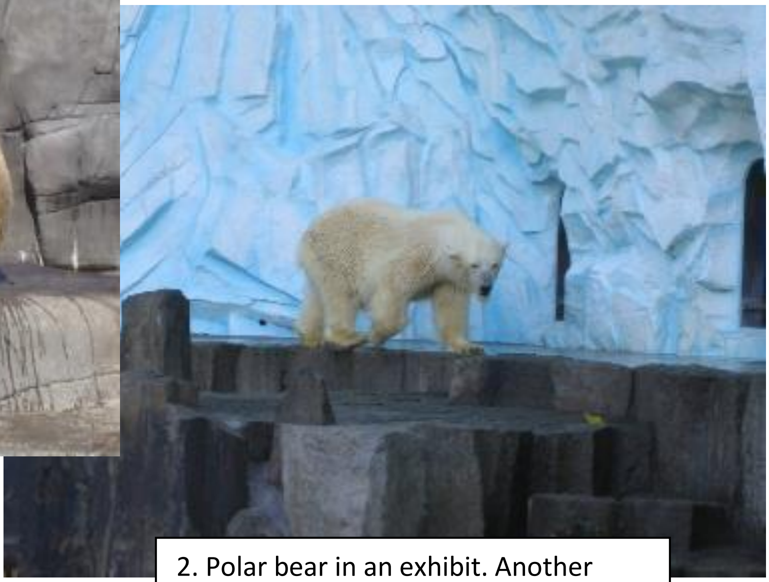

2. Polar bear in an exhibit. Another example of a stereotypy may be pacing back and forth repeatedly along a perimeter or within a certain space.

Other Behaviours

20. Feeding

In the case of feeding, if the bear is fed a seasonally variable diet according to the bear's behaviours and natural ecology, then the bear is given a welfare score of 3. If the bear's diet remains generally stable but may change once or twice a year, then it is given a welfare score of 2. If the bear is fed a standard basic diet throughout the year with no variability or fed to maintain a consistent bodyweight, then it is given a welfare score of 1.

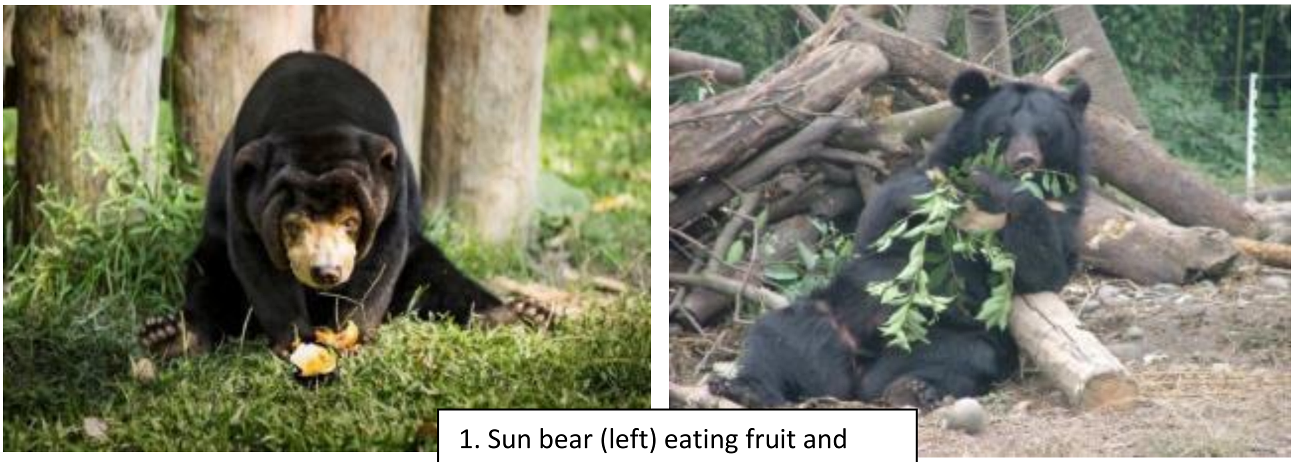

1. Sun bear (left) eating fruit and Asiatic black bear (right) eating vegetation.

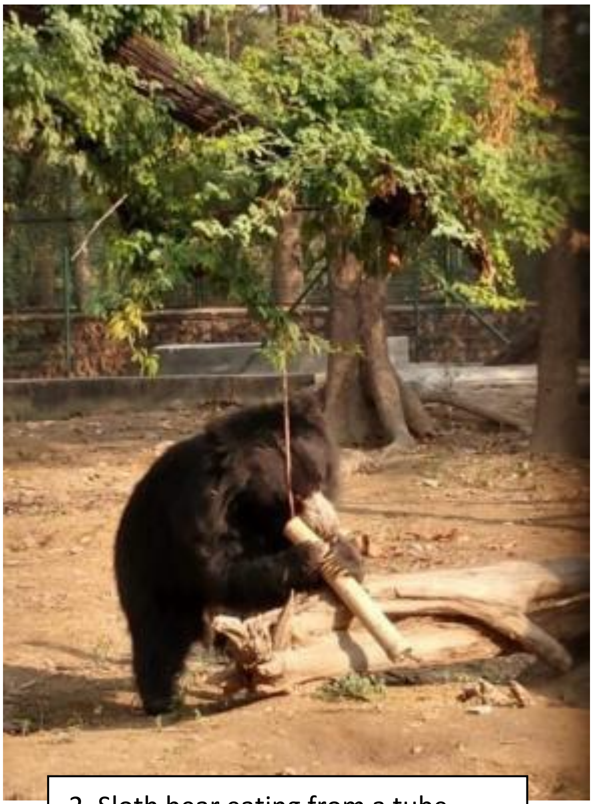

2. Sloth bear eating from a tube containing food.

21. Hibernatory/Winter Torpor Behaviours

This particular indicator only applies to temperate species of bear. If the bear has displayed seasonal dormancy/torpor and been encouraged to sleep through the winter, then it is given a welfare score of 3. If the bear has slowed down but only slept for short intermittent periods through the winter (e.g. the bear wakes up naturally on its own, stretches, walks around and then returns to den), then it is given a welfare score of 2. If the bear has slowed down but remained awake through the year, then it is given a welfare score of 1. Finally, if the bear has been routinely exhibited and not encouraged to sleep, then it is given a welfare score of 0.

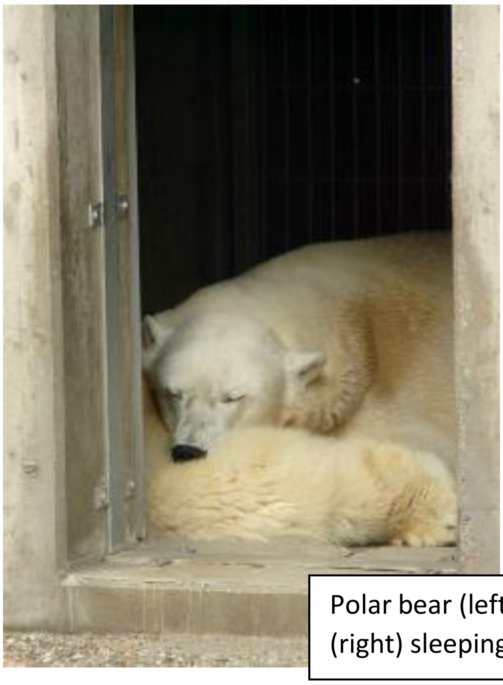

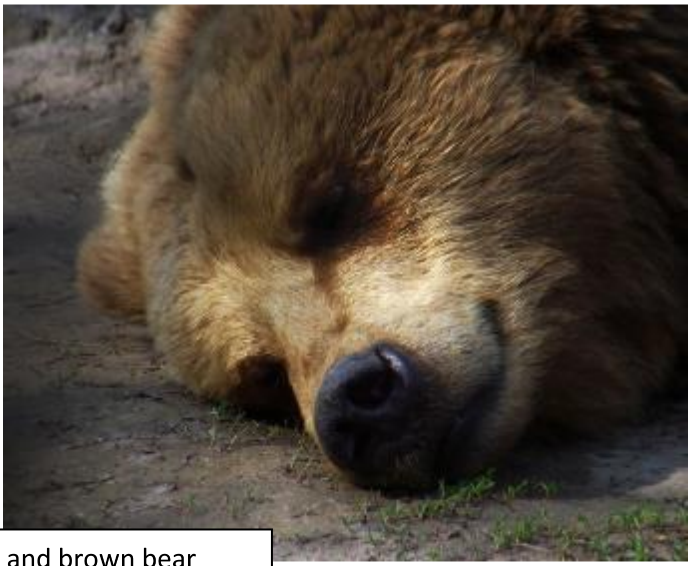

Polar bear (left) and brown bear (right) sleeping.

22. Environmental Enrichment Provision

Environment enrichment can be food-based (scatter feeding, ice-blocks, food-balls etc), provision of artificial or natural materials that are novel (new) to the bear, the addition of new objects for manipulation or playing, or olfactory (addition of scented materials, scent trails leading to food etc). Enrichment helps to encourage species-specific behaviours.

For this assessment, enrichment that has been incorporated into the bear's normal husbandry also classifies as enrichment. For instance, scatter feeding or hiding food within the environment, even if it is a daily exercise and part of normal husbandry, is considered enrichment. Also, if the bear is part of a positive reinforcement training program, this is also considered a form of cognitive enrichment.

If enrichment is provided daily by using an enrichment calender, the type of enrichment and the times of day it is provided are variable and/or the enrichment is considered part of daily husbandry or a training program, then it is given a welfare score of 3. If enrichment is provided 2 or 3 times a week, and the type of enrichment and the times it is provided are changed a few times a year, then it is given a welfare score of 2. If enrichment is provided less than twice a week, then it is given a welfare score of 1. If enrichment is never provided, then it is given a welfare score of 0.

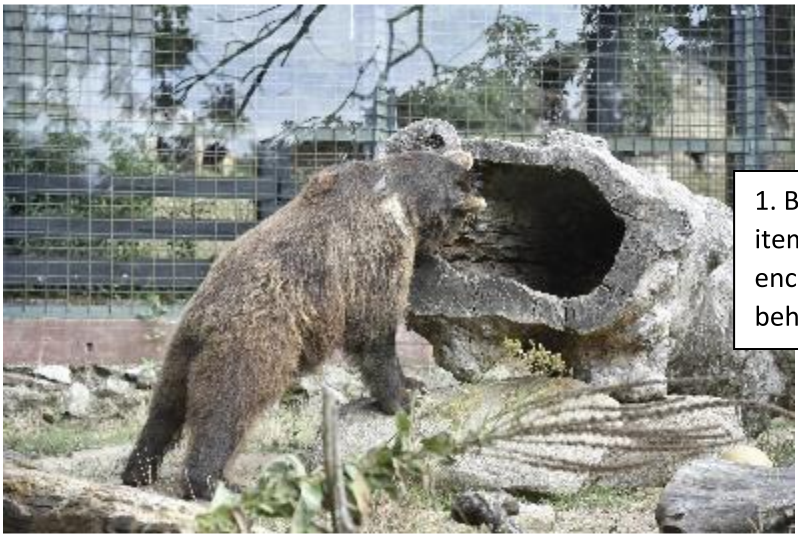

1. Bears can be led to hidden food items using scent trails. This encourages hunting and foraging behaviours.

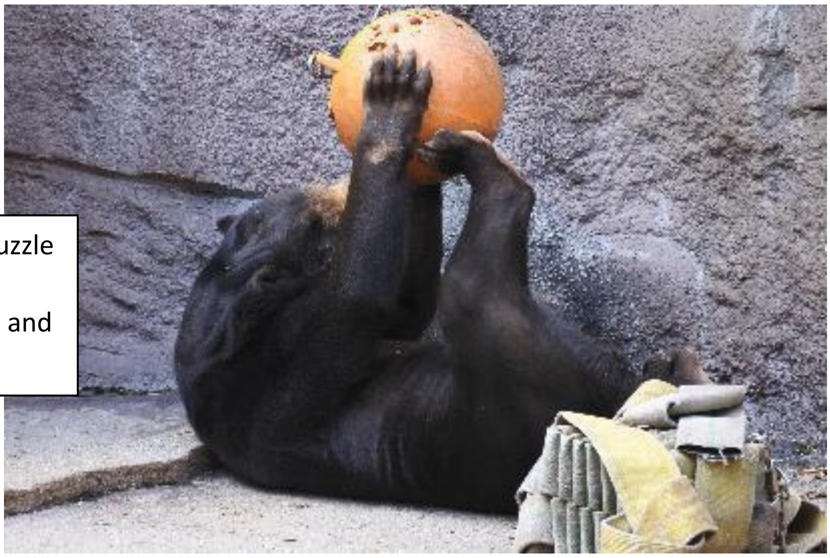

2. Bears can be provided with puzzle feeders (e.g. food-balls). This encourages foraging behaviours and provides cognitive enrichment.

Environmental

23. Access to Indoor and Outdoor Areas

Excluding routine enclosure servicing and cleaning, if the bear has access to both indoor and outdoor areas 24 hours a day, then it is given a welfare score of 3. If the bear has access to both areas, at the same time, for more than 18 hours a day then the bear is given a welfare score of 2. Finally, if the bear is locked into one area or its access is limited to one area for at least 7 hours a day, then it is given a welfare score of 1.

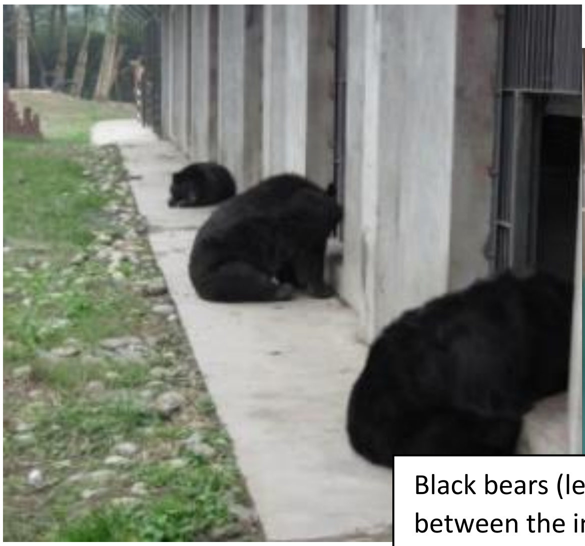

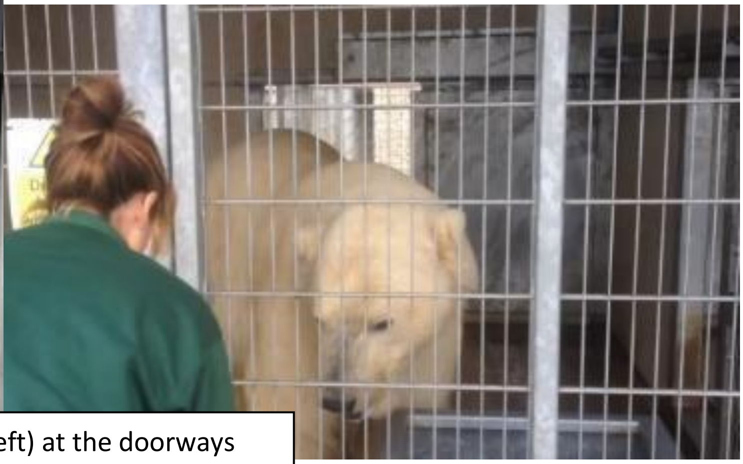

Black bears (left) at the doorways between the indoor and outdoor enclosures. Polar bear (right) in an indoor enclosure with slide open to outdoors.

24. Local Temperature Range/Climate

If the particular species is endemic to the local area or endemic to similar climatic conditions, then the bear is given a welfare score of 3. If the environment is broadly similar (temperate, arctic or tropical) to the species natural ecology, then it is given a welfare score of 2. If the environment is dissimilar/different to the species natural environment, then it is given a welfare score of 1.

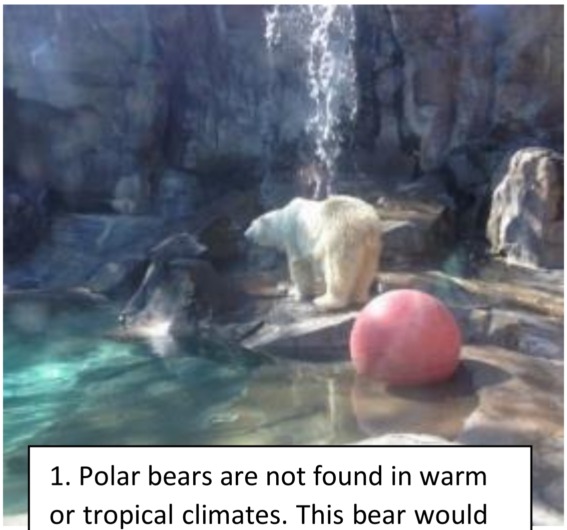

1. Polar bears are not found in warm or tropical climates. This bear would be given a welfare score 1.

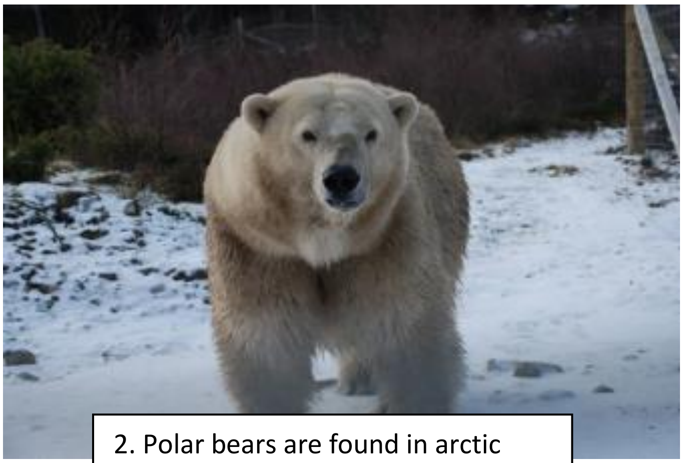

2. Polar bears are found in arctic climates. This polar bear would be given a score of 2 or 3 depending on the location of the zoo.

25. <u>Water Source for Bathing</u>

The water source, if it is provided, must be large enough for the bear to be able to lie down and bathe in. If a water source is present, it is given a welfare sore of 3. If a water source is absent or not deep enough, it is given a welfare score of 1.

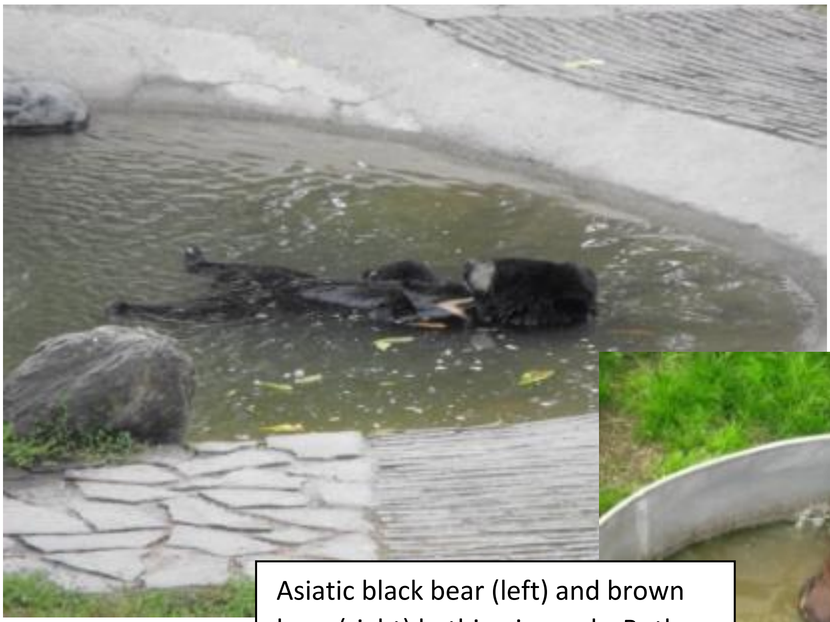

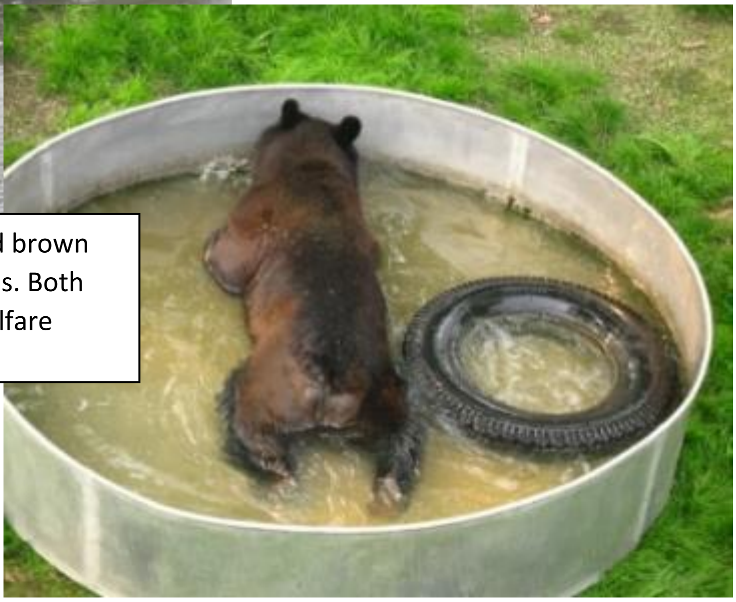

Asiatic black bear (left) and brown bear (right) bathing in pools. Both these bears are given a welfare score of 3.

26. Surfaces and Substrates

If the surfaces and substrates (e.g rocks, loose vegetation, hay, shredded paper etc) are naturalistic and varied to enable a range of behaviours including nesting, digging, bathing and foraging, then it is given a welfare score of 3. If the surfaces and substrates are naturalistic but behavioural opportunities are limited, then it is given a welfare score of 2. If the enclosure contains a mixture of artificial and natural surfaces (e.g. primarily artificial but with a digging pit and/or log piles), then it is given a welfare score of 1. If the enclosure is comprised of substrates or surfaces that cannot be manipulated and do not provide any behavioural opportunities, then it is given a welfare score of 0.

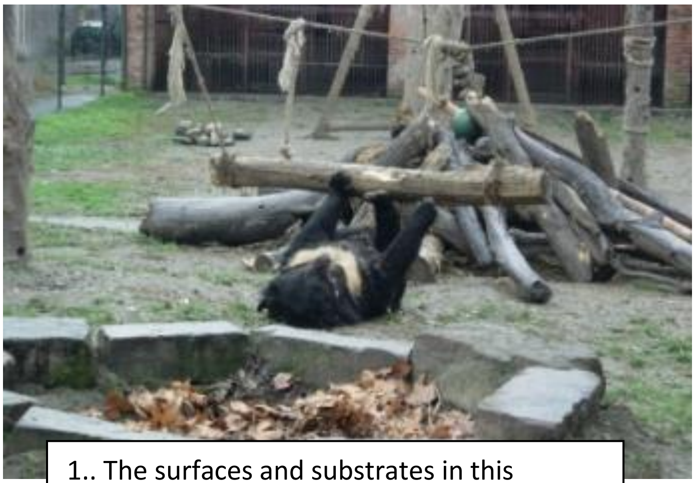

1.. The surfaces and substrates in this environment would be given a 3. There are natural substrates that allow the bear to dig, forage and nest.

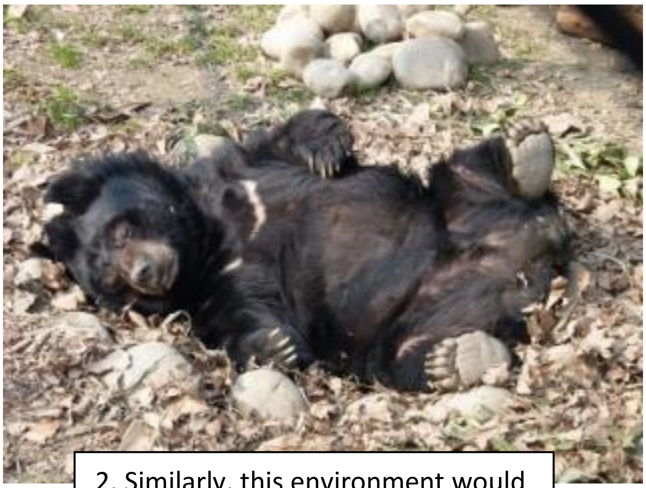

2. Similarly, this environment would be given a welfare score of 3 for the same reasons.

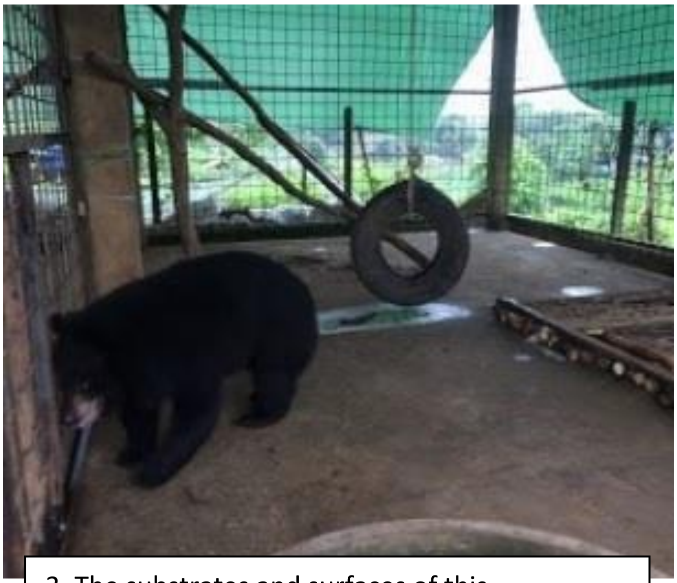

3. The substrates and surfaces of this environment would be given a score of 2. There are natural surfaces and substrates but the bear’s behavioural opportunities are limited.

27. View Out of Enclosure

If the majority of the enclosure consists of glass windows or fencing (or both) offering the bear the opportunity to view the surrounding area outside of the enclosure, then it is given a welfare score of 3. If the majority of the enclosure consists of high walls and the only 'open' area is utilised as a visitor viewing platform, then it is given a welfare score of 1.

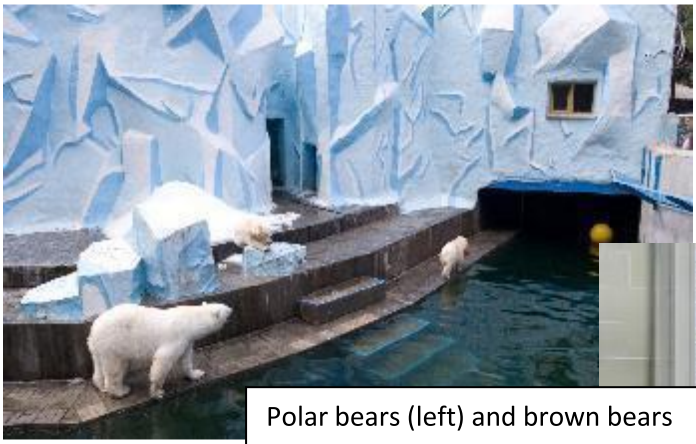
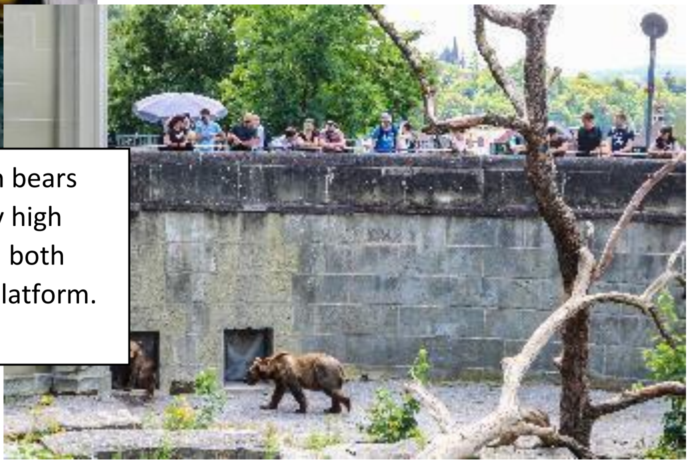

Polar bears (left) and brown bears (right) in pits surrounded by high walls. The only open area in both images is a visitor viewing platform. Welfare score of 1.

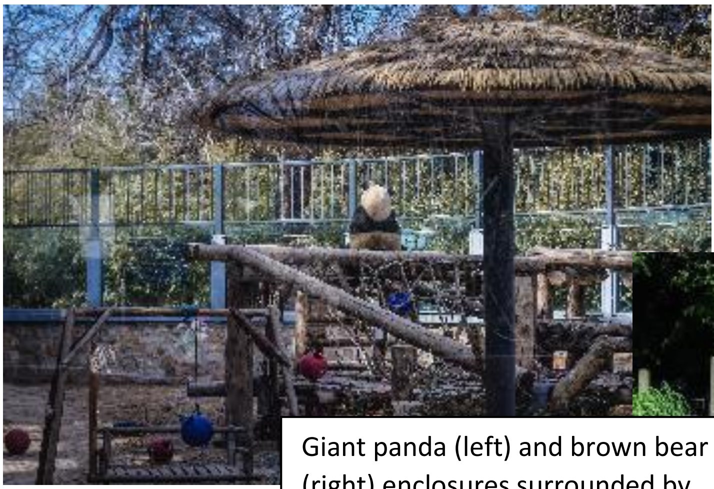
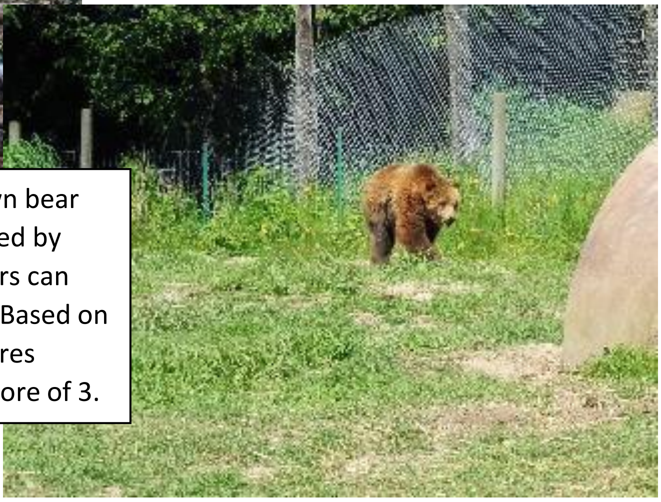

Giant panda (left) and brown bear (right) enclosures surrounded by fencing from which the bears can view the surrounding area. Based on these images, both enclosures would be given a welfare score of 3.

28. <u>Cover and Privacy</u>

If the enclosure offers varied topography, visual barriers and limited viewing to allow the bear to hide and avoid other bears and the public, it is given a welfare score of 3. If the enclosure offers some elements of privacy, it is given a welfare score of 2. If the animal is on view to visitors without cover or privacy, it is given a welfare score of 1.

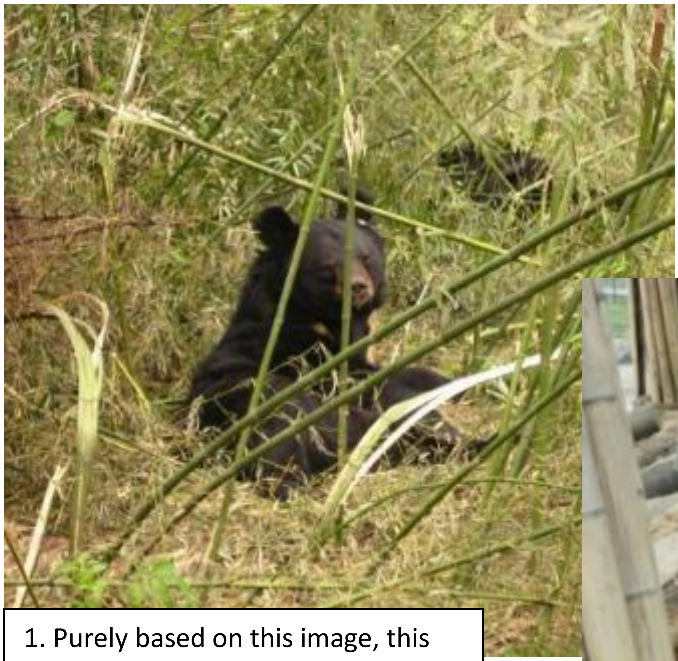

1. Purely based on this image, this enclosure would be given a welfare score of 2 as it offers some elements of privacy but could be improved.

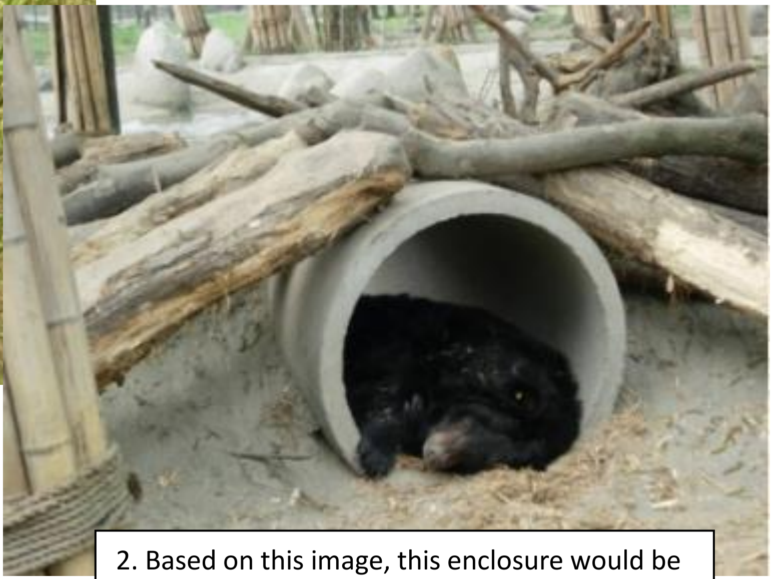

2. Based on this image, this enclosure would be given a welfare score of 3 as it offers visual barriers and varied topography.

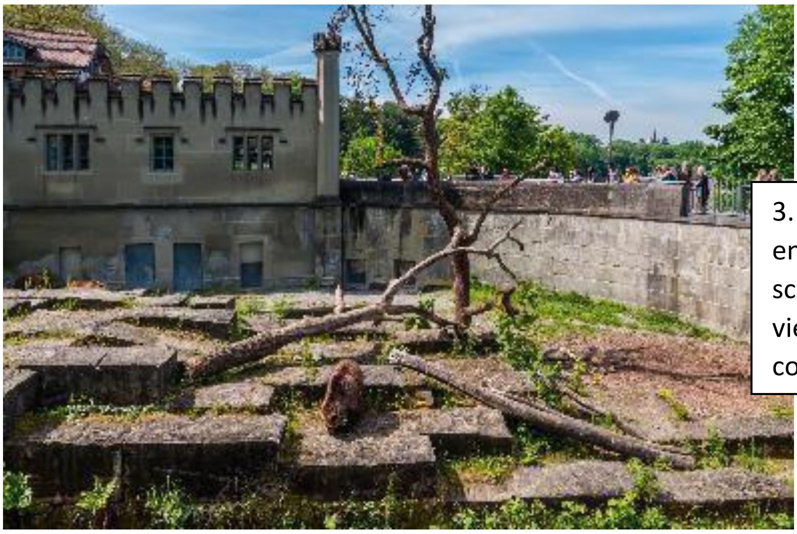

3. Based on this image, this enclosure would be given a welfare score of 1 as the enclosure can be viewed and the bear has little to no cover or privacy.

29. <u>Spatial Complexity</u>

If the enclosure offers environmental complexity and a range of behavioural opportunities including climbing, bathing, foraging, and exploration over a variety of 3D spaces, and there are enough resources to share among the social group, then it is given a welfare score of 3. If the enclosure offers some environmental complexity and some behavioural opportunities but this could be improved, then it is given a welfare score of 2. If the enclosure is simple with limited opportunities to display natural behaviours, then it is given a welfare score of 1. Finally, if the enclosure is barren with no complexity or variety, then it is given a welfare score of 0.

The welfare scores given in the images are purely based on what can be seen and inferred from the image.

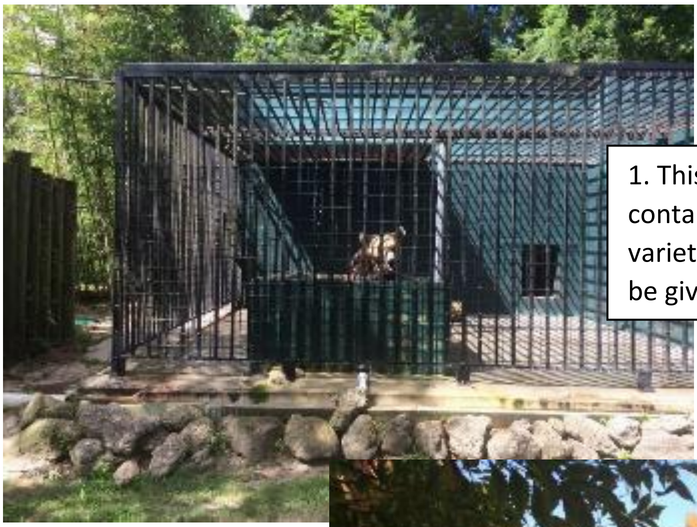

1. This enclosure is barren and contains no complexity or variety. This enclosure would be given a score of 0.

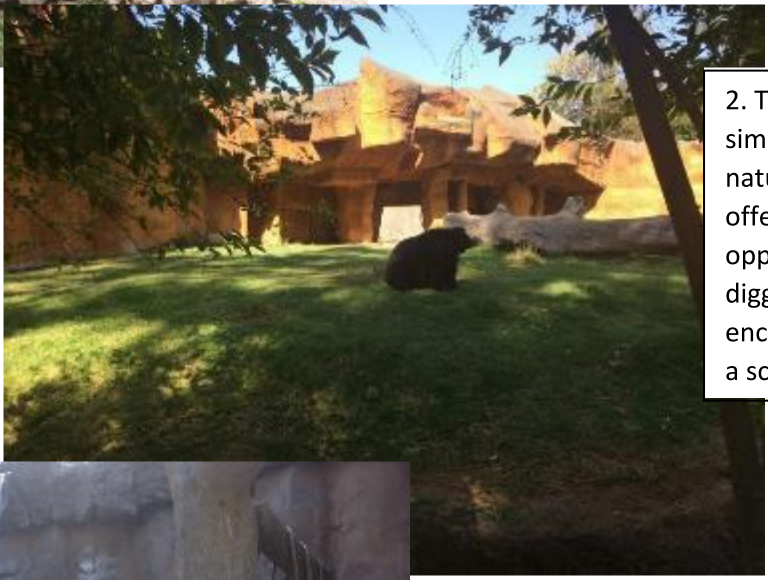

2. This enclosure is simple. It contains natural vegetation. It offers some behavioural opportunities including digging and foraging. This enclosure would be given a score of 2.

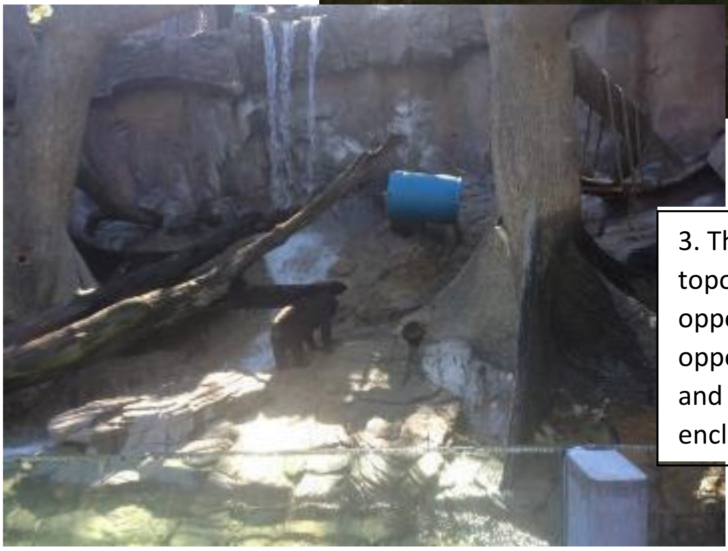

3. This enclosure has varied topography and offers climbing opportunities. Other behavioural opportunities (like digging, bathing, and nesting) are limited. This enclosure would score a 1.

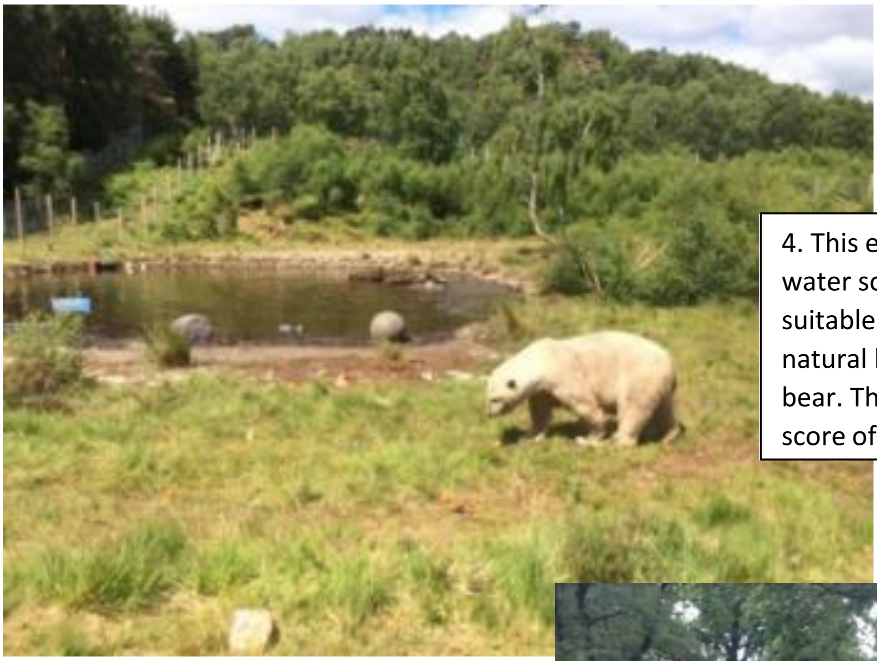

4. This enclosure is complex and contains a water source and natural vegetation suitable for foraging and is suitable for the natural behaviours and ecology of a polar bear. This enclosure would be given a score of 3.

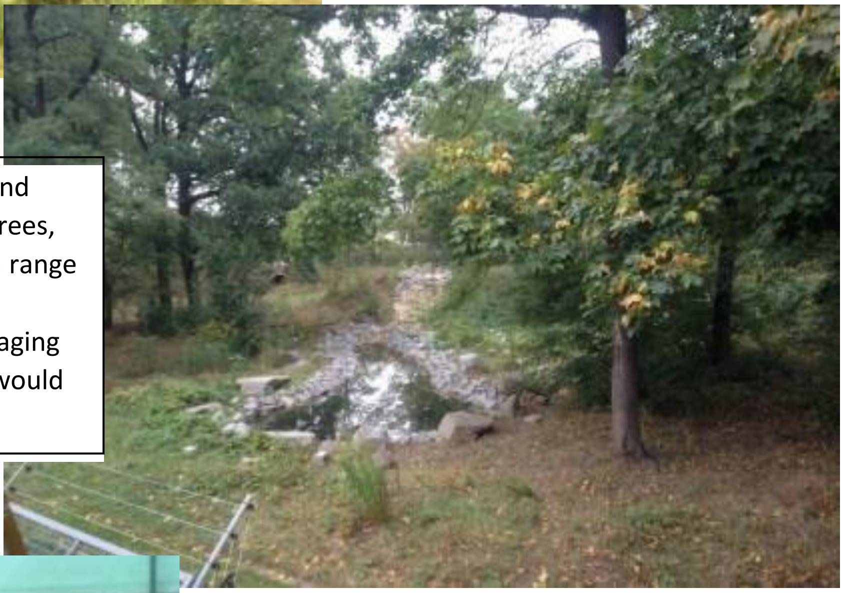

5. This enclosure is complex and contains natural vegetation, trees, and a water source. It offers a range of behavioural opportunities including digging, nesting, foraging and climbing. This enclosure would be given a score of 3.

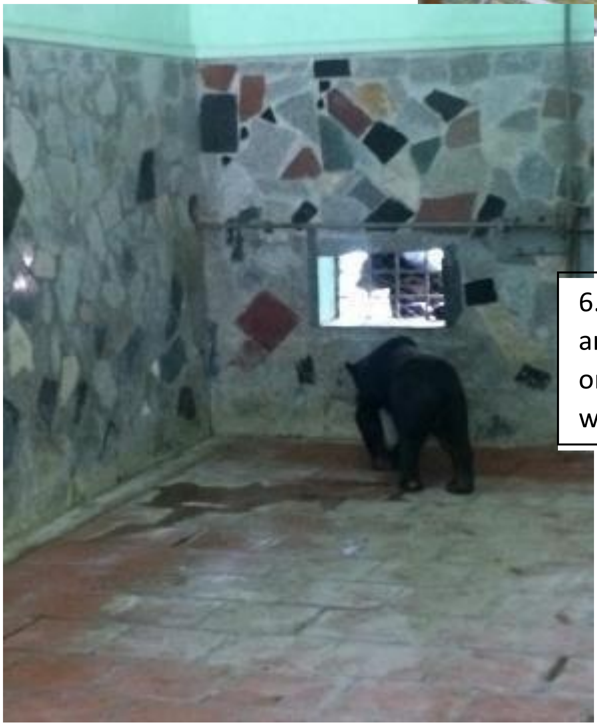

6. This enclosure is barren and offers no complexity or variety. This enclosure would score a 0.

Thank you for reading this training aid.

If you have any further questions, or anything is unclear, please feel free to contact me at hbacon@uclan.ac.uk

Thank you!

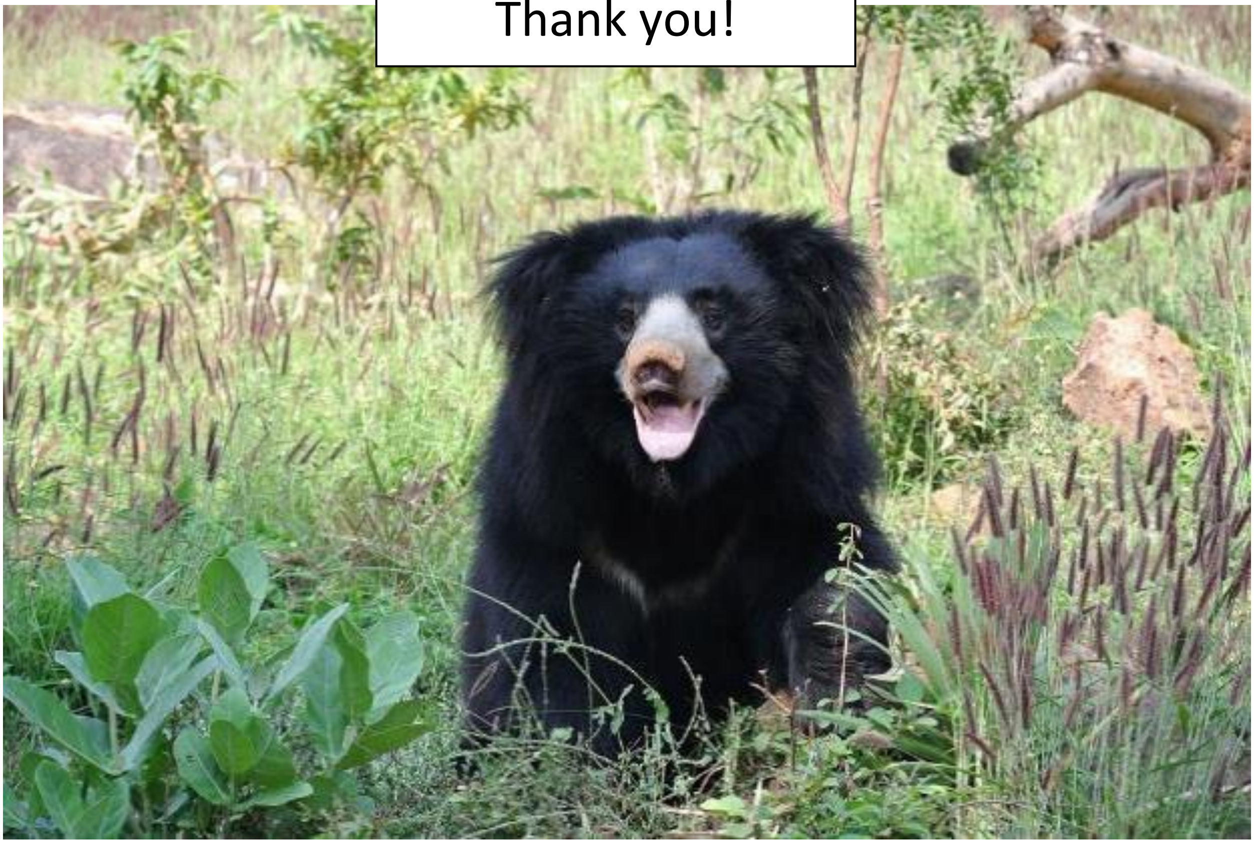

Supplement: Supplementary file 1 [file animals-11-03090-s001.zip › File_S2_Supplementary_Material.pdf]
